# Rapid inference of antibiotic susceptibility phenotype of uropathogens using metagenomic sequencing with neighbor typing

**DOI:** 10.1128/spectrum.01366-24

**Published:** 2024-11-29

**Authors:** Amanda C. Carroll, Leanne Mortimer, Hiren Ghosh, Sandra Reuter, Hajo Grundmann, Karel Brinda, William P. Hanage, Angel Li, Aimee Paterson, Andrew Purssell, Ashley Rooney, Noelle R. Yee, Bryan Coburn, Shola Able-Thomas, Martin Antonio, Allison McGeer, Derek R. MacFadden

**Affiliations:** 1 The Ottawa Hospital Research Institute, Ottawa, Ontario, Canada; 2 The Eastern Ontario Regional Laboratory, Ottawa, Ontario, Canada; 3 University of Freiburg 9174 , Freiburg, Germany; 4 Inria, Irisa, Univ. Rennes, Rennes, France; 5 Harvard T.H Chan School of Public Health, Harvard University, Cambridge, Massachusetts, USA; 6 Sinai Health 518775 , Toronto, Ontario, Canada; 7 The Ottawa Hospital, Ottawa, Ontario, Canada; 8 The University of Toronto, Toronto, Ontario, Canada; 9 University Health Network 7989 , Toronto, Ontario, Canada; 10 MRC Unit The Gambia at the London School of Hygiene and Tropical Medicine, Banjul, Gambia; 11 Department of Infection Biology, Faculty of Infectious and Tropical Diseases, London School of Hygiene & Tropical Medicine, London, United Kingdom; 12 Centre for Epidemic Preparedness and Response, London School of Hygiene & Tropical Medicine, London, United Kingdom; Central Texas Veterans Health Care System, Temple, Texas, USA

**Keywords:** antimicrobial resistance, metagenomics, nanopore, rapid diagnostics, genomics, urinary tract infection

## Abstract

Timely diagnostic tools are needed to improve antibiotic treatment. Pairing metagenomic sequencing with genomic neighbor typing algorithms may support rapid clinically actionable results. We created resistance-associated sequence elements (RASE) databases for *Escherichia coli* and *Klebsiella spp*. and used them to predict antibiotic susceptibility in directly sequenced (Oxford Nanopore) urine specimens from critically ill patients. RASE analysis was performed on pathogen-specific reads from metagenomic sequencing. We evaluated the ability to predict (i) multi-locus sequence type (MLST) and (ii) susceptibility profiles. We used neighbor typing to predict MLST and susceptibility phenotype of *E. coli* (64/80) and *Klebsiella spp*. (16/80) from urine samples. When optimized by lineage score, MLST predictions were concordant for 73% of samples. Similarly, a RASE-susceptible prediction for a given isolate was associated with a specificity and a positive likelihood ratio (LR+) for susceptibility of 0.65 (95% CI, 0.54–0.76) and 2.26 (95% CI, 1.75–2.92), respectively, with an increase in the probability of susceptibility of 10%. A RASE-non-susceptible prediction was associated with a sensitivity and a negative likelihood ratio (LR-) for susceptibility of 0.79 (95% CI, 0.74–0.84) and 0.32 (95% CI, 0.24–0.43) respectively, with a decrease in the probability of susceptibility of 20%. Numerous antibiotic classes could reasonably be reconsidered empiric therapy by shifting empiric probabilities of susceptibility across relevant treatment thresholds. Moreover, these predictions can be available within 6 h. Metagenomic sequencing of urine specimens with neighbor typing provides rapid and informative predictions of lineage and antibiotic susceptibility with the potential to impact clinical decision-making.

**IMPORTANCE:**

Urinary tract infections (UTIs) are a common diagnosis in hospitals and are often treated empirically with broad-spectrum antibiotics. These broad-spectrum agents can select for resistance in these bacteria and co-colonizing organisms. The use of narrow-spectrum agents is desirable as an antibiotic stewardship measure; however, it is counterbalanced by the need for adequate therapy. Identification of causative organisms and their antibiotic susceptibility can help direct treatment; however, conventional testing requires days to produce actionable results. Methods to quickly and accurately predict susceptibility phenotypes for pathogens causing UTI could thus improve both patient outcomes and antibiotic stewardship. Here, expanding on previous work showing accurate prediction for certain Gram-positive pathogens, we demonstrate how the use of RASE from metagenomic sequencing can provide informative and rapid phenotype prediction results for common Gram-negative pathogens in UTI, highlighting the future potential of this method to be used in clinical settings to guide empiric antibiotic selection.

## INTRODUCTION

Rising antimicrobial resistance (AMR) poses a major threat to public health globally (1, 2). Antibiotic resistance impairs our ability to adequately treat infections and is associated with increased morbidity and mortality (1). Although broad-spectrum antibiotic use could improve the reliability of coverage, it also promotes the selection of antibiotic resistance to these agents, which can lead to further spread in antibiotic-resistant organisms (AROs) (3). Rapid diagnostic approaches offer the promise of both improving the reliability of antibiotic coverage while also potentially narrowing the spectrum of initial antibiotic use. Despite this clear benefit, the advancement and clinical integration of rapid diagnostic methods for use in clinical care has been slow (4).

Our current methods of diagnosing infecting pathogens are still largely culture-based, which are both time- and resource-intensive (5). Healthcare providers must rely on empiric antibiotic therapy for the initial management of many infectious syndromes that carry the risk of either being unnecessarily broad spectrum or conversely inadequate for the underlying pathogen(s). Urinary tract infections (UTIs) have been significantly impacted by AMR, with the likelihood of activity for many first-line therapies declining over the decades due to rising AMR (6). This can have significant downstream effects on human health globally, given it is one of the most common community-acquired bacterial infections and can also manifest as severe disease including septic shock (7, 8). Urine specimens take approximately 36 h for the microbiology laboratory to complete pathogen identification and phenotypic antibiotic susceptibility testing, often following 12–24 h or more of transiting the specimen to a laboratory (9). From the time of collection, it can take up to 4 days for a patient with a UTI to receive their result (10). Delays in therapy, particularly in critically ill patients, can lead to increased morbidity and mortality, as well as patients receiving overly broad or ineffective antibiotics (11). Developing methods, which can give providers more actionable information sooner, could help improve their empiric therapies by identifying highly resistant organisms (in particular those resistant to first-line agents), as well as permitting the reconsideration of second-line antibiotics that had previously been abandoned due to low likelihood of activity (12).

Recently, much effort has been placed on sequencing-based methods for accurately assessing the microbial cause of illness and antibiotic susceptibility phenotypes with the aim of developing culture-free diagnostics (4, 13). However, these methods often rely upon lengthy- and resource-intense gene-based alignments to reference databases to identify potential resistance genes and profiles (4, 14, 15). Moreover, the presence or absence of a given resistance gene is insufficient to fully assess the resulting AMR phenotype, possibly through interactions with other resistance determinants, inducible resistance, or previously unrecognized mechanisms of resistance (16, 17). More recently, developments in *k*-mer-based strategies have shown that the analysis times required to match sequenced samples against curated databases may be greatly reduced (18). This method matches smaller portions of genetic material against *k-*mer databases made from the assemblies of known isolates with their associated antibiotic susceptibility profiles, meaning it can be made to run much faster than traditional gene-matching methods (18). The RASE method relies on neighbor typing algorithms, which predict both the (1) phylogroup (or sequence type) and (2) susceptibility patterns for unknown samples by utilizing the link between phylogeny and phenotype. Prior work has retrospectively evaluated the potential applications of RASE for *Streptococcus pneumoniae* and *Neisseria gonorrhoeae* (19, 20) but has not been prospectively evaluated for the most common Gram-negative pathogens *Escherichia coli* and *Klebsiella spp*.

Pairing sequencing of primary urine specimens with fast neighbor typing algorithms could be used to rapidly generate information about a pathogen’s likely antibiotic susceptibility phenotype to guide empiric treatment until the availability of gold-standard culture-based results. Urine samples are particularly well suited for this approach, given the often high bacterial loads present and the potential for impacting current care practices.

In this multicenter prospective study, we demonstrate the use of ONT MinION direct metagenomic sequencing, paired with *k*-mer-based neighbor typing to rapidly predict the multilocus sequence type (MLST) and susceptibility profile of typical uropathogen(s) identified in suspected urinary tract infection.

## RESULTS

### Reference database generation

**Fig 1:**
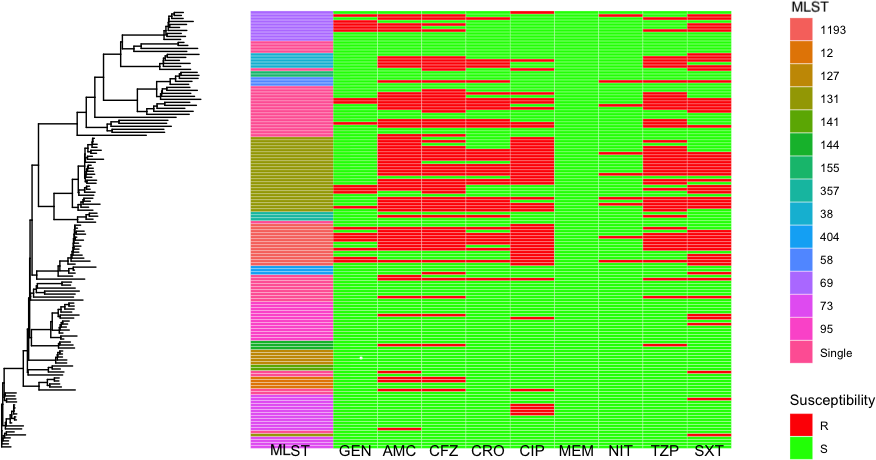
Combined genetic tree (mashtree), with heatmap showing the variety of ST, and susceptibility results for the database isolates for *E. coli*.

**Fig 2:**
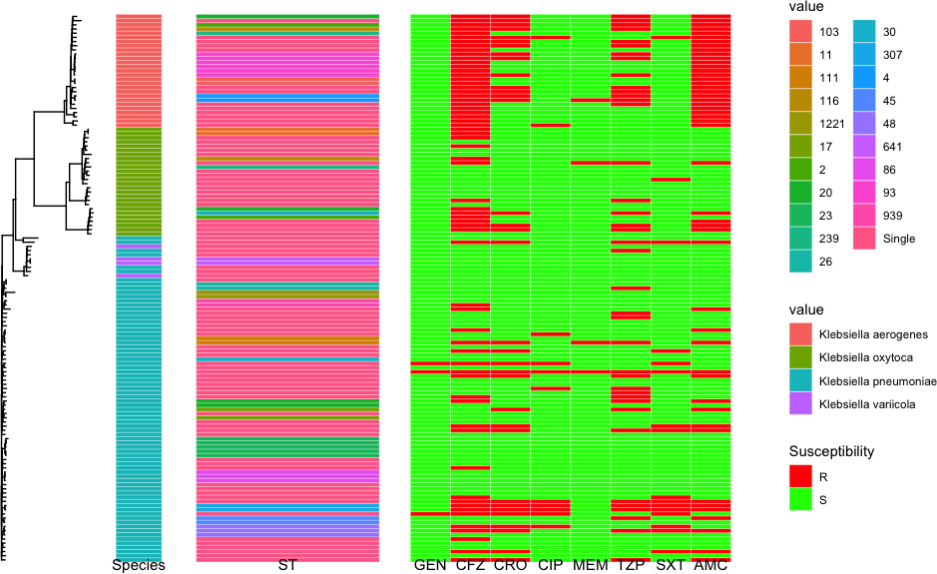
Combined genetic tree (mashtree), with heatmap showing the variety of ST, and susceptibility results for the database isolates for *Klebsiella spp*. ST belonging to *K. pneumoniae*, *K. aerogenes*, *K. oxytoca*, and *K. variicola* are denoted as *P*#, A#, O#, and V#, respectively.

We first sought to develop regional reference databases of clinical isolates from ICUs in two large cities in Ontario for two Gram-negative pathogens of concern. We collected 131 (*Klebsiella spp*. including *Klebsiella pneumoniae*, *Klebsiella variicola*, *Klebsiella oxytoca*, and *Klebsiella aerogenes*) and 148 (*E. coli*) isolates for these databases (Table 1). These isolates were collected from urine samples that were culture-positive for one of the pathogens of interest whether they had resistance to any specific agent; these isolates, therefore, represent a sampling of the ICU population at the time of collection from August 2020 to January 2021. There were 106 multilocus sequence types (MLST) represented in the *Klebsiella* spp. database (58 *K*. *pneumoniae*, 25 *K*. *oxytoca*, 20 *K*. *aerogenes*, and 3 *K*. *variicola*), with 51 represented in the *E. coli* data set (Table 1). Commonly encountered *E. coli* MLSTs included those consistent with *E. coli* lineages known to cause a large proportion of human extraintestinal infections (21, 22). As expected, genetic trees for each reference set show clustering of related isolates by MLST (Fig. 1 and 2).

**TABLE 1:** Reference database and test sample paired isolate characteristics from samples collected in Ontario, Canada, including sample number, unique multilocus sequence types (MLSTs), and phenotypic susceptibility profiles for the evaluated antibiotics^*a*^

				% Susceptible per antibiotic								
Sample type	Organism	Samples	Distinct MLSTs represented	GEN	AMC	CFZ	CRO	CIP	MEM	NIT	TZP	SXT
Regional reference database	*E. coli*	148	51	88	59	60	74	65	100	94	68	64
	*Klebsiella spp*.	131	99	98	64	50	65	89	97	-	63	88
Test samples	*E. coli*	64	24	91	45	45	70	55	100	98	67	72
	*Klebsiella* spp.	16	13	94	81	81	81	81	100	-	81	75

^*a*^ For each organism type, we included in our database susceptibility to commonly used antibiotics; however, due to limitations in clinical testing, not all clinically available antibiotics could be included. GEN = gentamicin; AMC = amoxicillin-clavulanic acid; CFZ = cefazolin; CRO = ceftriaxone; CIP = ciprofloxacin; MEM = meropenem; NIT = nitrofurantoin; TZP = piperacillin-tazobactam; SXT = trimethoprim-sulfamethoxazole.

### ONT metagenomic sequencing

We sequenced metagenomic DNA from remnant primary urine specimens from ICUs in Toronto and Ottawa with ONT MinION. Following initial processing with Kraken2, we ran the RASE algorithm with the organism-specific regional reference database for *E. coli* and a genus-specific regional reference database for *Klebsiella spp*. based on the dominant uropathogen. Stability was achieved with a median of 203 (Q1-Q3: 161–345) reads for *E. coli* and 260 (Q1-Q3: 169–559) reads for *Klebsiella spp*.. Time to stability occurred at a median of 9 (Q1-Q3: 5–28) and 19 (Q1-Q3: 7–114) min of sequencing time for *E. coli* and *Klebsiella spp*., respectively (Table S1). The median retained reads belonging to the putative pathogen of interest were 3281 (Q1-Q3: 2740, 3573) for *E. coli* and 3340 (Q1-Q3: 2186–3359) for *Klebsiella spp*., corresponding to approximately 82% and 83.5% reads, respectively (Table S1). We had 88 initial metagenomic samples (69 *E. coli* and 19 *Klebsiella spp*.), of which 8 (9.1%) were removed, as they had dominant pathogens (by read count) that were discordant with the selected clinical uropathogen (by culture), and these were removed from the subsequent analysis (Table S2). Three samples (two with cultures yielding *E. coli* and one with cultures yielding Klebsiella spp.) had a non-uropathogen as dominant, but the expected uropathogen was the second most common contributor, and these reads were retained. Repeated taxonomic classification, of only reads available up to the time of stability, confirmed consistency with dominant pathogen classification and ensured reproducible classification could be made prospectively. All subsequent analyses were performed on all retained reads classified as the dominant pathogen only, unless otherwise specified.

### Predicting multilocus sequence type (MLST)

We next assessed the MLST of the closest match predicted by RASE and compared it with the presumed true MLST (determined from the short-read sequence data from the isolates derived from the matching primary urine samples) (Table 2; Table S3). The samples were classified using the MLST package (23, 24). The correct prediction of sequence type by RASE was improved when stratifying by lineage score, which marks greater confidence in the best match lineage predictions (Table 2). A lineage score (LS) is provided for each call, with higher scores indicating increased confidence in lineage assignments, and any stratification for LS was done using LS >0.5. Concordance, the match between true and predicted MLST, for *E. coli* samples when the lineage score was greater than or equal to 0.5% was 76.2% compared with 40.9% with LS below 0.5. Concordance for *Klebsiella spp.* was 55.6% when the lineage score was greater than or equal to 0.5 compared with 14.2% with LS below 0.5. Further investigation found that low LS occurred more frequently when the corresponding MLST is absent in the database and thus may mediate discordant results (Table S3), with the exception of a few sequence types, such as ST1193 of *E. coli*, where the MLST is well-represented in the database (*n* = 14), but five of the six samples with MLST 1193 had LS <0.5.

**TABLE 2:** Multilocus sequence type (MLST) prediction for primary urine specimens^*a*^

			Lineage score (LS) <0.5		Lineage score (LS) ≥0.5	
Organism type	Total number of samples (n)	Number of unique MLSTs	Concordant (%)	Not concordant (%)	Concordant (%)	Not concordant (%)
*E. coli*	64	24	40.9 (9)	59.1 (13)	76.2 (32)	23.8 (10)
*Klebsiella spp*.	16	13	14.2 (1)	85.7 (6)	55.6 (5)	44.4 (4)

^*a*^ Concordant: the predicted MLST for the best match from RASE matches the true MLST. Not concordant: the predicted MLST for the best match does not match the true MLST.

### Predicting antibiotic susceptibility phenotypes

**Fig 3:**
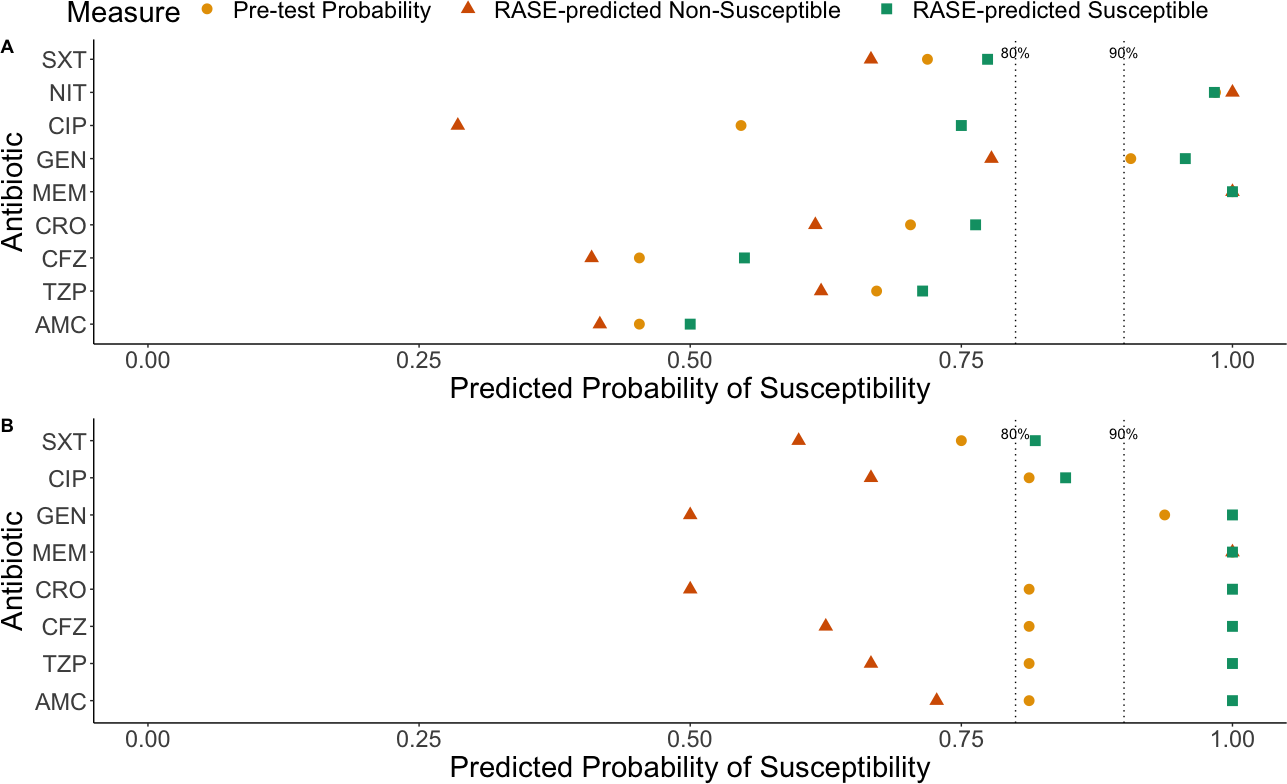
Pre-test probability (yellow circles), RASE-predicted non-susceptible (orange triangles), and RASE-predicted susceptible (green squares) values determined for the overall prediction for each tested antibiotic for (**A**) *E. coli* and (**B**) *Klebsiella spp*. Dotted lines at 0.8 and 0.9 represent the low-severity and high-severity disease thresholds, respectively (25), derived from (10). A threshold of 80% probability of susceptibility has been previously established as acceptable for guiding treatment in patients with low- to moderate-severity infection. A threshold of 90% probability of susceptibility has been found to be acceptable for guiding treatment in patients with high-severity infections. Antibiotic short forms used in figure: AMC = amoxicillin-clavulanic acid; TZP = piperacillin-tazobactam; CFZ = cefazolin; CRO = ceftriaxone; MEM = meropenem; GEN = gentamicin; CIP = ciprofloxacin; NIT = nitrofurantoin; SXT = trimethoprim-sulfamethoxazole.

To assess the ability to predict AMR, we computed the pre-test probability of susceptibility (or prevalent susceptibility) and the probability of true susceptibility for isolates predicted to be resistant or susceptible when using RASE (Fig. 3). Values higher than the pre-test probability and/or the thresholds of 80%–90% indicate that RASE predictions for susceptibility are more likely to be truly susceptible. Overall likelihood ratios for RASE-prediction for all antibiotic susceptibilities in each of *E. coli* and *Klebsiella spp*. are shown in Table 3, whereas the details of sensitivity, specificity, and negative and positive likelihood ratios for each antibiotic-pathogen pair are shown in Table S4. The pre-test (or prevalent) probabilities of susceptibility vary widely for *E. coli* (Fig. 3A), depending on the antibiotic, and are generally less susceptible, with more variation than for *Klebsiella spp*. (Fig. 3B). The probability of susceptibilities given either RASE-predicted non-susceptible or RASE-predicted susceptible are generally left and right of the pre-test probability, respectively, as expected. This is summarized in the pooled (overall) data, via sensitivity and specificity values as well as likelihood ratios (which are independent of baseline prevalence of resistance) of susceptibility. We found (without stratification) a sensitivity of 0.69 (95% CI: 0.64, 0.74) and a specificity of 0.65 (95% CI: 0.56, 0.73) as well as a positive likelihood ratio (LR+) of 1.96 (95% CI: 1.60, 2.40) and a negative likelihood ratio (LR-) of 0.48 (95% CI: 0.39, 0.58) (Table 3) for predicting susceptibility. We also assessed the change in probability of susceptibility, given a RASE prediction of susceptible or non-susceptible (Table 3). For the pooled data, the pre-test probability was 0.74, and the absolute change in predicted susceptibility for RASE-predicted non-susceptible isolates was a reduction of 16.6%, whereas the absolute change in susceptibility for RASE-predicted susceptible was an increase of 10.9%. RASE-predicted susceptibilities values were also evaluated to see if they were moved over relevant clinical treatment thresholds representing acceptable likelihood of susceptibility for low-severity (80%) and high-severity (90%) disease (25); in this case, the value for trimethoprim-sulfamethoxazole for *Klebsiella spp.* was moved above the 90% threshold. Notably, numerous agents (gentamicin, ciprofloxacin, piperacillin/tazobactam, ceftriaxone, and cefazolin) for both organism types had their probabilities of susceptibilities moved below typical treatment thresholds when there was a RASE-predicted non-susceptible prediction, which shows the potential value for identifying infections at high likelihood of resistance that may necessitate different antibiotic therapies. We also assessed if there were improvements to prediction using RASE with either local databases (Ottawa- and Toronto-specific), or a European (EuSCAPE) database, to determine if assessing region-specific samples to region-specific databases improved analysis or if broader, although geographically different, databases improved prediction. In the case of the local databases, these did not clearly perform any better than using the regional databases for both *E. coli* and *Klebsiella spp*. (Fig. S2; see Supplementary Results for expanded analysis). Using the European database produced similar results to the regional databases (Fig. S3) and yielded MLST concordance for some samples that had an MLST not represented in the regional databases (Table S8).

**TABLE 3:** Test characteristics for predicting susceptibility by different stratification conditions for each organism type and pooled^*a*^

			Pathogen-of-interest only reads							
						Mean				
Organism type	Total susceptibilities	Stratified by	Susceptibilities lost (n, %)	Sensitivity	Specificity	LR+ (95% CI)	LR- (95% CI)	Pre-test probability	Absolute decrease in probability of susceptibility with RASE-predicted non-susceptible (%)	Absolute increase in probability of susceptibility with RASE-predicted susceptible (%)
*E. coli*	576	None	0 (0%)	0.69 (0.63,0.74)	0.63 (0.53,0.72)	1.84 (1.50, 2.28)	0.50 (0.40, 0.61)	0.71	−16.0	10.8
		SS	97 (16.8%)	0.76 (0.71,0.81)	0.57 (0.46,0.68)	1.77 (1.44, 2.18)	0.42 (0.33, 0.54)	0.73	−27.5	13.6
		LS	225 (39.1%)	0.8 (0.74,0.85)	0.64 (0.53,0.76)	2.22 (1.70, 2.90)	0.32 (0.23, 0.43)	0.71	−19.7	9.65
		SS + LS	288 (50%)	0.87 (0.82,0.92)	0.58 (0.44,0.73)	2.09 (1.59, 2.73)	0.23 (0.15, 0.34)	0.73	−34.9	11.9
		Concordant	207 (35.9%)	0.7 (0.63,0.77)	0.75 (0.65,0.84)	2.77 (1.99, 3.86)	0.40 (0.31, 0.52)	0.70	−21.6	16.7
		Discordant	369 (64.1%)	0.67 (0.58,0.76)	0.38 (0.16,0.59)	1.07 (0.84, 1.36)	0.88 (0.62, 1.25)	0.74	−2.4	1.28
*Klebsiella spp*.	128	None	0 (0%)	0.71 (0.61,0.81)	0.8 (0.6,1)	3.56 (1.47, 8.63)	0.36 (0.21, 0.61)	0.84	−18.4	10.7
		SS	8 (6.25%)	0.76 (0.66,0.86)	0.8 (0.6,1)	3.8 (1.57, 9.19)	0.3 (0.17, 0.53)	0.83	−21.1	9.28
		LS	56 (43.75%)	0.77 (0.65,0.89)	0.73 (0.42,1.04)	2.83 (1.07, 7.49)	0.32 (0.15, 0.67)	0.85	−23.3	11.7
		SS + LS	64 (50%)	0.87 (0.77,0.97)	0.73 (0.42,1.04)	3.18 (1.21, 8.40)	0.18 (0.07, 0.45)	0.83	−36.2	11.1
		Concordant	80 (62.5%)	0.65 (0.46,0.84)	0.73 (0.42,1.04)	2.38 (0.88, 6.42)	0.48 (0.23, 1.01)	0.77	−15.2	11.8
		Discordant	48 (37.5%)	0.75 (0.63,0.86)	0.89 (0.67,1.11)	6.72 (1.05, 42.85)	0.29 (0.13, 0.61)	0.89	−19.5	9.40
Pooled	704	None	0 (0%)	0.69 (0.64,0.74)	0.65 (0.56,0.73)	1.96 (1.60, 2.40)	0.48 (0.39, 0.58)	0.74	−16.7	10.9
		SS	105 (14.9%)	0.87 (0.83,0.9)	0.6 (0.5,0.7)	1.91 (1.55, 2.34)	0.40 (0.32, 0.50)	0.75	−26.6	12.9
		LS	281 (39.9%)	0.79 (0.74,0.84)	0.65 (0.54,0.76)	2.26 (1.75, 2.92)	0.32 (0.24, 0.43)	0.73	−20.4	10.0
		SS + LS	352 (50%)	0.87 (0.82,0.91)	0.6 (0.47,0.73)	2.18 (1.68, 2.83)	0.22 (0.15, 0.32)	0.75	−35.2	11.7
		Concordant	287 (40.8%)	0.69 (0.63,0.76)	0.75 (0.66,0.84)	2.73 (1.99, 3.73)	0.41 (0.32, 0.53)	0.71	−20.9	16.2
		Discordant	417 (59.2%	0.69 (0.62,0.76)	0.45 (0.27,0.64)	1.26 (0.99, 1.61)	0.68 (0.50, 0.94)	0.78	−7.19	3.69

^*a*^ SS: Susceptibility score. LS: Lineage Score. SS+LS: Susceptibility and lineage score combined. Concordant: the predicted MLST for the best match from RASE matches the true MLST. Discordant: the predicted MLST for the best match does not match the true MLST.

### Improving phenotype prediction using confidence scores

We assessed whether RASE-produced lineage and susceptibility scores could be used to improve prediction. The lineage score (LS) is provided for each call, with higher scores indicating increased confidence in lineage assignments. Susceptibility scores (SS) are provided for each antibiotic: scores range from 0 to 1, with lower values representing higher confidence for non-susceptible calls, and higher values representing higher confidence for susceptible calls; values between 0.4 and 0.6 represent calls where RASE is less confident that the phenotype prediction is in fact resistant or susceptible.

Stratifying by SS (SS <0.4 and SS >0.6), LS (LS >0.5), or both resulted in the loss of data for increasing percentages of antibiotic susceptibility calls. Although stratification by SS seemed to offer no obvious advantage, there was a suggestion of possible benefit of stratification by lineage score (Table 3). Sensitivities were higher for all levels of stratification, with values increasing the least when stratifying by SS and increasing the most with combined stratification by LS and SS. However, specificities were all the same or lower following stratification (Table 3). Thus, stratification by SS or LS has the potential to improve sensitivity, but not specificity.

We also assessed the performance when accounting for concordance of predicted and actual MLST. We hypothesized that the potential benefit of LS stratification might be mediated through the concordance of MLST. Although there was a trend suggesting increased accuracy of prediction for MLST concordant *E. coli*, this was not observed for *Klebsiella spp*. For *E. coli*, concordance improved LR + and LR- compared with discordant calls, but this was not observed with *Klebsiella spp*. (Table 3). Note that the pooled results are largely informed by *E. coli*, but this reflects the common relative distribution of the two pathogens in UTI. Similarly, concordance improved both the sensitivity and specificity for *E. coli* but had variable impacts for *Klebsiella spp*. (Table 3).

**Fig 4:**
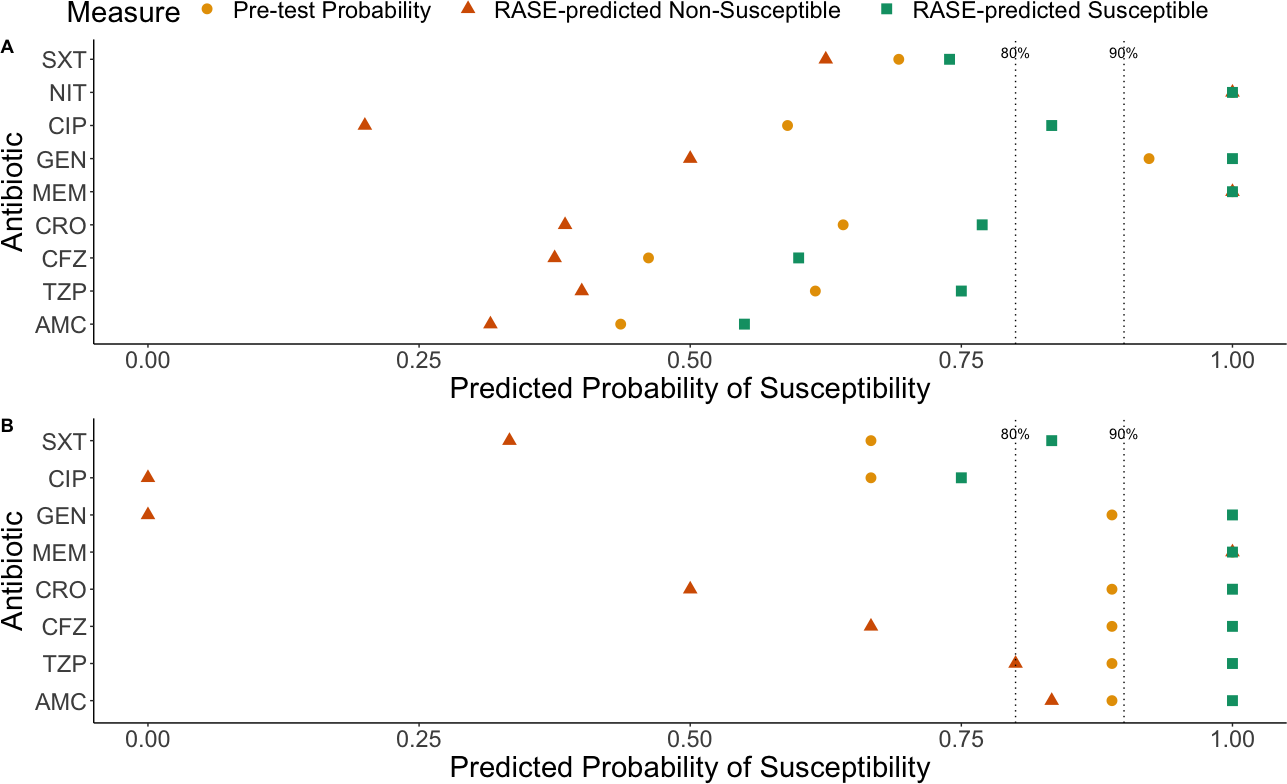
Pre-test probability (yellow circles), RASE-predicted non-susceptible (orange triangles), and RASE-predicted susceptible (green squares) values determined for the overall prediction for each tested antibiotic for (**A**) *E. coli* and (**B**) *Klebsiella* spp. when stratifying by lineage score (LS) >0.5. A threshold of 80% probability of susceptibility has been previously established as acceptable for guiding treatment in patients with low- to moderate-severity infection. A threshold of 90% probability of susceptibility has been found to be acceptable for guiding treatment in patients with high-severity infections. Antibiotic short forms used in figure: AMC = amoxicillin-clavulanic acid; TZP = piperacillin-tazobactam; CFZ = cefazolin; CRO = ceftriaxone; MEM = meropenem; GEN = gentamicin; CIP = ciprofloxacin; NIT = nitrofurantoin; SXT = trimethoprim-sulfamethoxazole.

Although conclusions based on changes in test characteristics by stratification are limited by the size of the confidence intervals (Table 3), we can compare the impact on point estimates (Fig. 4). For *E. coli*, using the LR point estimates and comparing with a non-stratified approach, the LS stratification has the potential to support reconsidering of one additional (and commonly used) antibiotic across the 80% susceptibility threshold (ciprofloxacin). For *Klebsiella spp*. RASE-predicted susceptible probabilities were marginally improved with stratification, particularly for ceftriaxone (Fig. 4B). However, this did not reflect a major change in LR+ (Table 3). For *E. coli* and *Klebsiella spp*., we also separated the antibiotics by whether they were typically administered orally or intravenously and calculated the relevant test characteristics (Fig. S1). For *E. coli*, the RASE-predicted susceptible probability moved to 80% or greater for antibiotics typically administered intravenously (improving potential for empiric use) or orally. In *Klebsiella spp*., the RASE-predicted susceptible probability for orally administered antibiotics was also moved over the 80% threshold, whereas those typically administered intravenously were moved over the 90% threshold.

## DISCUSSION

In this study, we evaluate a rapid diagnostic approach of paired metagenomic sequencing with neighbor typing to predict MLST and antibiotic susceptibility phenotype across two important Gram-negative pathogens (19) in urine samples. Using this approach, we were able to improve the confidence of empiric antibiotic susceptibility predictions over the local antibiogram. In particular, we demonstrate that this method has the potential to support the reconsideration of clinically relevant but less commonly used antibiotics for use prior to definitive phenotype identification by moving their likelihood of activity above treatment thresholds and identifying infections with a high likelihood of non-susceptibility that would require broader spectrum antimicrobials.

Prior studies using metagenomic approaches for rapid antibiotic resistance prediction have shown that susceptibility and non-susceptibility can be inferred within 10 min of sequencing for both *Streptococcus pneumoniae* and *Neisseria gonorrhoeae* using the RASE algorithm (18). Other work linking metagenomic approaches to antibiotic resistance prediction has included mapping reads to specific genomes or collections of antibiotic resistance genes and showed some success (26–28). However, these methods often require matching to specific genes or rely on unsupervised approaches, and linking genotype to phenotype can be challenging (29). Methods using machine learning and artificial intelligence are also on the rise, which also works to link genotypic information with phenotypic data, and these methods have shown some success in predicting AMR phenotypes (30–33). With this study, we show neighbor typing combined with metagenomic sequencing has the potential to provide rapid and informative predictions of antibiotic resistance in the two most common Gram-negative pathogens, *E. coli* and *Klebsiella*.

The approach used in this paper is rapid with necessary sequencing times of typically less than 30 min. We can determine the expected total workflow duration based on these sequencing times as well as the typical sample processing and library preparation times. Using the methods described herein, it typically takes 90 min to pre-process and deplete eukaryotic DNA, 60 min to extract prokaryotic DNA, and 3 h for library preparation. Taken together, this means results can be available within 6 h of sample collection (excluding transit times). Of this total time period, the first 5.5 h require some degree of hands-on time, although this could be reduced (34–36). One present limitation lies in the specialized training and familiarity with bioinformatics workflows needed for completing and interpreting analyses. However, these processes can be automated and simplified for ease of use. As well, the requirement of batching samples to achieve economy of scale is a limitation to the rapidity of analyses. However, this could be overcome through the use of the Flongle system (ONT), which can be used as a single sample disposable platform at a similar cost per run to minION flow cells (a single Flongle flow cell currently costs approximately one–tenth of the price of a single standard flow cell).

Information that can significantly improve empiric antibiotic selection (currently based on local antibiograms) can be imperfect yet still benefit decision-making (22). Although microbiologic diagnostics have historically produced dichotomized results (e.g., sensitive vs. resistant), recognizing that even these have their own critical limitations, new technological advances support the use of alternative data streams that can provide probabilistic predictions at faster timelines (14, 37). Further work is needed to find how this information can be best incorporated into clinical decision-making.

We evaluated how stratification by two RASE-produced metrics might improve the confidence in calls made by RASE. Although not statistically significant, stratification by SS, LS, or LS + SS may provide some improvement to the predictions for both *E. coli* and *Klebsiella spp*., with some benefit to likelihood ratios, sensitivities, and specificities, but at a cost of significant loss of the proportion of specimens with predictions. In keeping with this, for *E. coli,* concordance improved as LS improved, which fits with phylogroup-based prediction and supports the notion that improving the ability to identify a related strain within the reference database (in this case of the same MLST) improves the ability to predict antibiotic susceptibility. Although a perfect relationship between MLST and relatedness is not expected, MLST (using the traditional seven loci) has reliably been used for the identification of genus, species, and lineage/clonal complexes (38). However, for the distinction between highly related clones or strains, a higher resolution is necessary to distinguish relatedness and may require other classification methods (38, 39). Interestingly, for *Klebsiella spp*. this did not clearly hold. Although neighbor typing for *Klebsiella spp*. improved calls for susceptibility/non-susceptibility, it may be independent of MLST, though it is hard to definitively conclude this with the existing sample size for this genus.

This method of neighbor typing and antibiotic susceptibility prediction fundamentally relies on the databases that are used for making these predictions. Our results using a European (EuSCAPE) database showed that performance was similar to that of the regional database, whereas local databases seemed to perform no better, meaning that in this case, databases from other continents could be used to make predictions. This shows that the physical proximity of database samples may matter less than having a diverse reference database and having samples that are genetically proximate. Combined with the results from the EuSCAPE database, this highlights the need for large, well-structured reference databases with a diversity of isolates to make better concordant matches.

There are several strengths to the approach outlined in this paper. The proposed method can be fast, with the potential for informative results to be generated in as little as 6 h. Rapid turnaround of samples shows this approach could meaningfully impact on empiric management of infections, with the potential to improve patient outcomes and reduce the reliance on broader-spectrum antibiotics. Moreover, this method could be adaptable for other approaches beyond susceptibility prediction, including rapidly ruling out potential transmission. Finally, based on our evaluation using an international database, we show that this method may also be implementable globally with large databases.

There are limitations to the work we present here. First, most of our results are based on a single geographic region (Ontario, Canada), and further study will be required to determine utility elsewhere. We also have a limited number of samples presented here, and only from two genera of *Enterobacterales*; hence, our results are underpowered for detecting statistically significant (or insignificant) differences in the test characteristics following stratification. Also, this method currently cannot quantify the pathogen for significant growth, as we are unable to relate sequencing results to the quantitation of colony-forming units (CFU). Moreover, 9.1% of our remnant urine samples were excluded from the analysis because the expected uropathogen was not present. This could produce inconsistencies between what was reported in the culture report and the true sample and could fail to detect a pathogen that was present in the sample at low titers. However, these limitations reflect ongoing challenges with the diagnosis of UTIs using culture-based techniques (9). Finally, one concern of the RASE method is how it accounts for mobile genetic elements, such as plasmids. AMR genes are commonly found on plasmids in *E. coli* and *Klebsiella spp.* (40). Presently, RASE is unable to distinguish if there is a presence or absence of plasmids in a genome, as well as if there has been any plasmid recombination or partial integration with the chromosome. However, whether plasmids are present is still relevant to the RASE method. That is, some lineages of bacteria are better at accepting and retaining plasmids due to the impact that plasmid acquisition can have on the fitness of the host (41). If lineages have been well-sampled, there should be lineages captured with and without AMR plasmids with resistance phenotypes, and this risk of resistance should be reported by the susceptibility score produced by RASE (18). Although the presence or absence of AMR plasmids is not explicitly accounted for in this work, sampling of various lineages, including several isolates from the same lineage, should capture a broad view of samples with and without plasmids and should assess some level of the risk for resistant phenotypes from this sampling.

In conclusion, we show that combining metagenomic sequencing with neighbor typing algorithms has the potential to support rapid and informative predictions of the susceptibility of dominant pathogens present in urine samples. Current prediction performance would benefit from future optimization to further improve potential clinical impacts. Additional work is needed to evaluate approaches to accurately quantify absolute bacterial load in metagenomic specimens and prospectively evaluate impacts on UTI treatment.

## MATERIALS AND METHODS

### Study design

**Fig 5:**
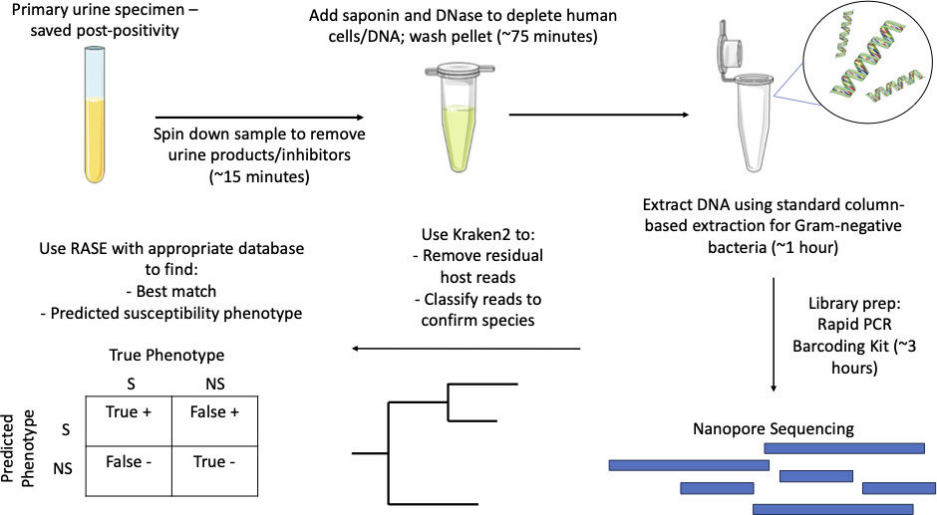
Sample preparation, sequencing, and bioinformatics workflow overview.

We performed a multicenter prospective study evaluating the role of rapid metagenomic sequencing combined with *k*-mer-based neighbor typing approaches for the purposes of rapidly predicting antibiotic resistance from clinical urine samples (Fig. 5). First, we prospectively generated RASE *k*-mer organism type reference databases for two Gram-negative pathogens isolated from critical care patients (*E. coli* and *Klebsiella spp*.). Database isolates were collected from samples submitted to clinical microbiology laboratories from critical care units at each of four participating tertiary care hospitals (two in Toronto and two in Ottawa) in Ontario, Canada. Subsequently, from the same four critical care units, we prospectively collected remnant urine specimens containing *E. coli* or *Klebsiella spp*. as the dominant Gram-negative in culture and sequenced them using the Oxford Nanopore MK1c. RASE was then used to predict multilocus sequence type (MLST) and antibiotic susceptibility phenotype. The study was approved by the Ottawa Hospital Science Network (#20200108–01H), as well as the University Health Network (#20–5677) and the Sinai Health (20–0161-E) Research Ethics Boards in Toronto, with a waiver of consent from all institutions.

### Database generation

*E.coli* and *Klebsiella spp*. isolates from clinical specimens (from any anatomical source) originating from critically ill patients were collected between August 2020 and March 2021 from the four participating Ontario hospitals and stored at −70°C until nucleic acid extraction. Draft whole genome sequences were generated using short-read approaches (Illumina or MGI), and clinically derived antibiotic susceptibility phenotypes were collected as meta-data. Assembled sequences along with antibiotic susceptibility phenotype and genotypic multi-locus sequence typing (MLST) (23, 24, 42) were used to generate the RASE *k*-mer reference databases (18). For each of *E. coli* and *Klebsiella spp.,* we constructed a regional database (combination of Toronto and Ottawa isolates). We primarily evaluated the performance of prediction for samples collected from both locations against these regional databases. However, although not the main focus of our investigation, we also performed additional analyses (described below) evaluating the performance of local databases (i.e., using reference databases from the same city to make predictions) and the use of an international (European) database.

### Characterization of uropathogens from primary urine specimens

Between May 2021 and September 2022, remnant urine specimens from critical care patients submitted to the microbiology laboratory for bacterial culture that were growing a single Gram-negative morphotype of interest (*E.coli* or *Klebsiella spp*.) in clinically significant amounts, regardless of the Gram-positive growth, were retrieved and stored at −70°C until nanopore sequencing. Additionally, the Gram-negative bacterial isolates corresponding to the primary specimens of interest were stored at −70°C, and whole genome sequencing was subsequently performed using short-read approaches (Illumina or MGI), and clinically derived antibiotic susceptibility phenotypes were collected as meta-data.

### Pathogen identification and antibiotic susceptibility testing

For both reference isolates and uropathogen isolates from primary urine specimens, pathogens were identified to the species level by MALDI-TOF. Antibiotic susceptibility testing was performed as per standard clinical evaluation at their source hospital laboratory. Antibiotic susceptibility testing was performed using a combination of disc diffusion testing, and commercial MIC methods (BD Phoenix M50 [Ottawa], and Vitek 2 [Toronto]) and throughout this period 2020 CLSI breakpoints were used.

### ONT metagenomic sequencing

Stored remnant urine samples were thawed, and mechanical host cell depletion was performed, followed by DNA extraction and Qubit quantitation. Libraries were prepared using ONT’s Rapid PCR Barcoding Kit with multiplexing. Nanopore metagenomic sequencing was performed on the ONT MinION MK1c sequencer using R9.4.1 flow cells with 11 specimens and a process negative control (12 samples total), for a 24-h run time. Basecalling and subsequent demultiplexing were automatically done using the onboard base calling software guppy (6.4.6). See Fig. 3 for an overview of the method.

### Metagenomic bioinformatic analyses

After sequencing was completed, de-multiplexed raw read files (fastqs) were selected, and read quality measures output by the ONT software were reviewed. The fastq files containing the first 4000 MinION-generated reads were analyzed using a *k*-mer-based taxonomic classification system, Kraken2 (version 2.1.3), with standard RefSeq database (k2_standard_20230605) (43), and only prokaryotic reads were retained for storage and analysis. The Kraken2 generated classification report was used to identify the dominant bacterial pathogen in the sample, which was then paired with KrakenTools (44) to filter for pathogen-specific reads, which were used to generate these data. The classification reports also informed selection of the appropriate RASE reference database, which was then used to run the RASE pipeline (version 0.1.0.0; please see Supplementary for detailed usage of RASE). All analyses were run with the Mac OS X operating system.

### Assigning RASE prediction stability

Prior to defining the MLST and susceptibility predictions made by RASE, we needed to define the point at which the prediction appeared stable. The lineage score (LS) was developed as a potential indicator of lineage prediction confidence, and thus, we chose this to establish a stable prediction (18). Specifically, we defined stability as the point where the LS did not fluctuate more than a magnitude of 0.1 for 100 consecutive reads. The overall read count assigned at the initial time of sequencing was used, which includes both unfiltered and filtered reads, and therefore would facilitate the establishment of a typical range of reads required to achieve a stable prediction that could be applied prospectively.

### Predicting antibiotic susceptibility phenotype and MLST

Antibiotic susceptibility predictions were evaluated for amoxicillin-clavulanic acid, cefazolin, ceftriaxone, ciprofloxacin, gentamicin, meropenem, nitrofurantoin, piperacillin-tazobactam, and trimethoprim-sulfamethoxazole. Standard clinical phenotypic testing of the urine specimen isolate was used as the outcome and was classified as either susceptible (S) or non-susceptible (NS) (which includes both resistant and intermediate susceptibility results). RASE-predicted susceptibility results were directly compared with the clinical phenotype result for the relevant antibiotics for each sample. Test characteristics including sensitivity, specificity, prevalence, positive predictive value (PPV), and negative predictive values (NPV) of susceptibility were calculated (see Supplementary Materials and Methods for further details). PPV was used to indicate the probability of susceptibility if RASE predicted susceptible. 1-NPV was used to generate the probability of susceptibility if RASE predicted non-susceptible. Improvements to susceptible and non-susceptible predictions are indicated by moving these values right and left of the pre-test probability, respectively. We compared these values with previously established thresholds for the probability of susceptibility found to be acceptable to guide treatment in patients with low- to moderate-severity infection (80%) and high-severity infection (90%) (10). These values were compiled for various combinations of antibiotics and organism types, and 95% confidence intervals of test characteristics were also generated. Final values were then visualized using ggplot2 (45), using R version 4.3.0 (46). We generated Bayesian figures of the prevalence, PPV, and 1-NPV values for each of the antibiotics of interest for each organism type (22).

### Evaluating the impact of reference database geographic scales

Although the aim of our study was to evaluate regional reference databases for MLST and antibiotic susceptibility phenotype prediction, we also evaluated the predictive performance of local (city-specific) databases and a larger international collection (Supplementary Methods) for predicting MLST and antibiotic susceptibility phenotype. For the latter, we used EuSCAPE isolates, which were collected in hospitals across Europe for the purpose of AMR surveillance (47, 48).

See Supplementary Methods for full details on all described methods.

## SUPPLEMENTAL MATERIAL

Figure S1 spectrum.01366-24-s0001.tiff Test characteristics for *E. coli* and *Klebsiella* spp. for antibiotics grouped by route of administration.

Figure S2 spectrum.01366-24-s0002.tiff Some test characteristics for *E. coli* following assessment with local versus regional databases.

Figure S3 spectrum.01366-24-s0003.tiff Test characteristics for *Klebsiella pneumoniae* samples assessed using the EuSCAPE RASE database.

Supplemental material spectrum.01366-24-s0004.docx Figures S1 to S3, Tables S1 to S8, and supplemental methods and results.

ASM does not own the copyrights to Supplemental Material that may be linked to, or accessed through, an article. The authors have granted ASM a non-exclusive, world-wide license to publish the Supplemental Material files. Please contact the corresponding author directly for reuse.
