# Supplementary figures and images for: Rapid inference of antibiotic susceptibility phenotype of uropathogens using metagenomic sequencing with neighbor typing
Source: Microbiol Spectr. 2024 Nov 29;13(1):e01366-24. doi: 10.1128/spectrum.01366-24 (PMC11705937; doi:10.1128/spectrum.01366-24)

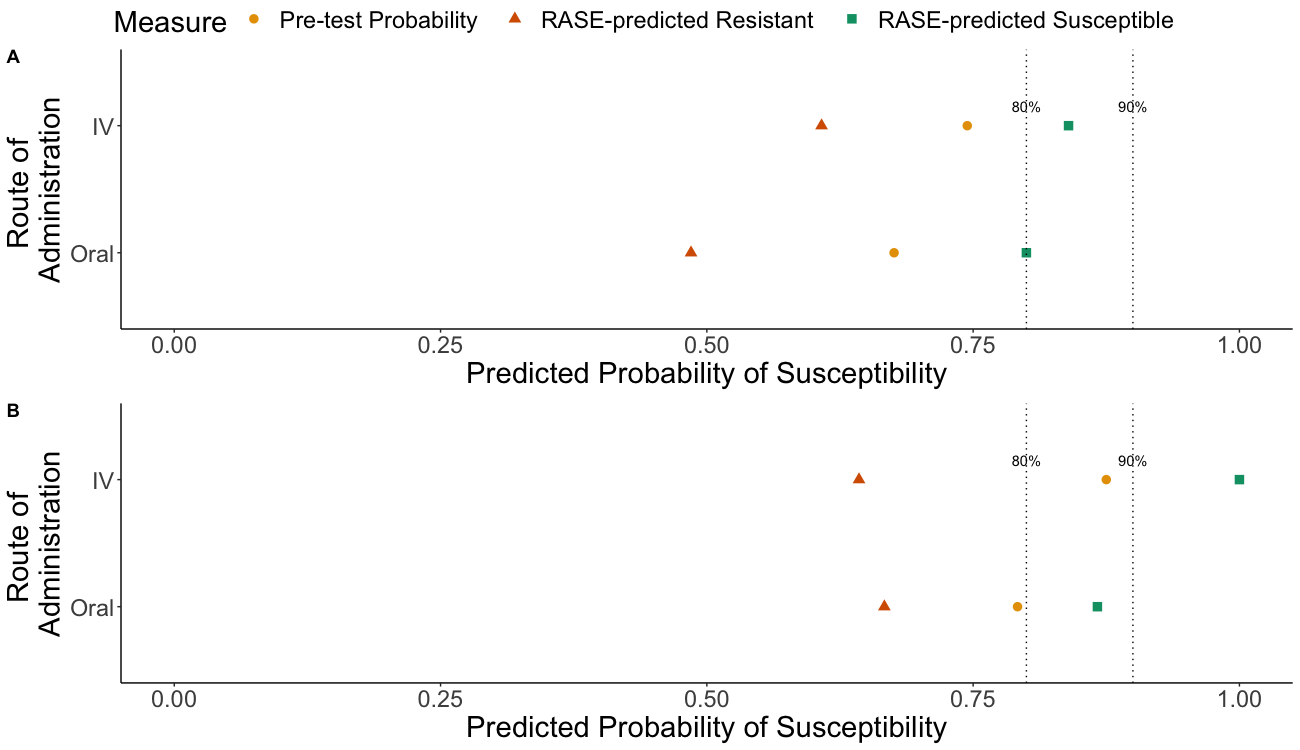

Supplement: Figure S1 — Test characteristics for E. coli and Klebsiella spp. for antibiotics grouped by route of administration. [file spectrum.01366-24-s0001.tiff]

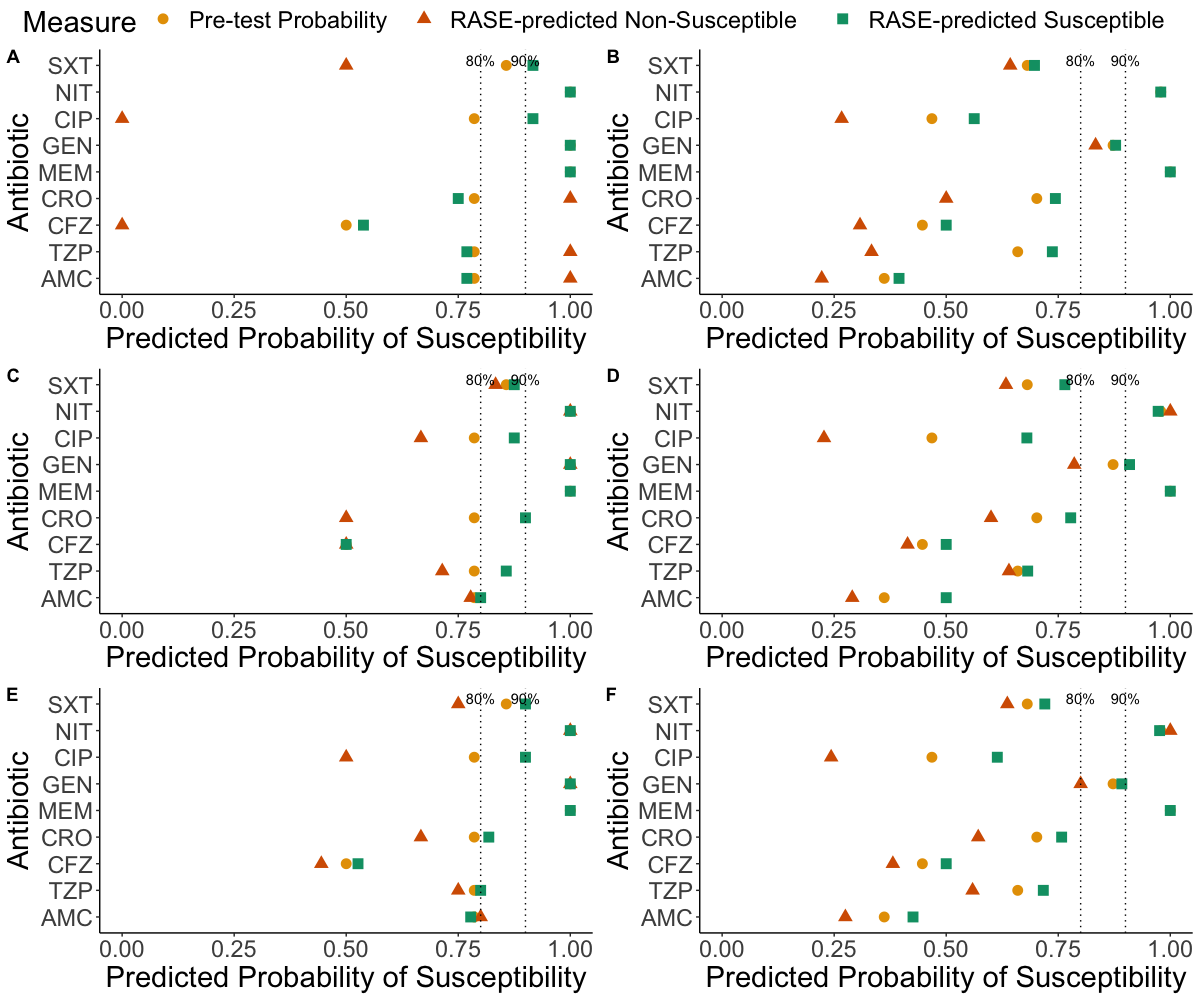

Supplement: Figure S2 — Some test characteristics for E. coli following assessment with local versus regional databases. [file spectrum.01366-24-s0002.tiff]

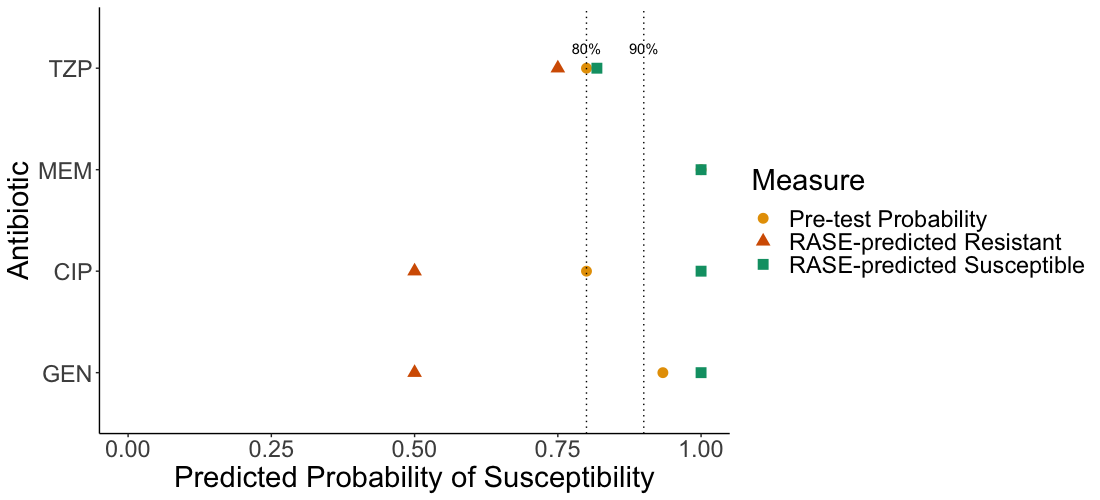

Supplement: Figure S3 — Test characteristics for Klebsiella pneumoniae samples assessed using the EuSCAPE RASE database. [file spectrum.01366-24-s0003.tiff]
