# Supplementary material for: Rapid inference of antibiotic susceptibility phenotype of uropathogens using metagenomic sequencing with neighbor typing
Source: Microbiol Spectr. 2024 Nov 29;13(1):e01366-24. doi: 10.1128/spectrum.01366-24 (PMC11705937; doi:10.1128/spectrum.01366-24)
Supplement: Supplemental material — Figures S1 to S3, Tables S1 to S8, and supplemental methods and results. [file spectrum.01366-24-s0004.docx]

**SUPPLEMENTAL TABLES**

Table S1: Sequencing summary results for metagenomic urine samples for the first 4000 reads generated by MinION sequencing, as well as at the time of stability.

| Organism Type | Number of Samples Analyzed (n) | Median Prokaryotic Reads* | Median Pathogen Reads* | Median Prokaryotic Reads at Stability | Median Pathogen-specific Reads at Stability* | Median Time to Stability (min)* | Median Read Length (bp)* | Samples with high non-uropathogen reads | Samples removed due to other predominant uropathogen |
| --- | --- | --- | --- | --- | --- | --- | --- | --- | --- |
| *E. coli* | 64 | 3938 (3550, 3972) | 3281 (2740, 3573) | 227 | 203 (161, 345) | 9 (5, 28) | 3450 (2441, 4645) | 2 | 5 |
| *Klebsiella spp.* | 16 | 3838 (3025, 3982) | 3142 (1208, 3382) | 323 | 260 (169, 559) | 19 (7, 114) | 3706 (2664, 4940) | 1 | 3 |

* Numbers presented in brackets for these values indicate the interquartile range (Q1, Q3).

Table S2: Primary urine samples containing the presence of other uropathogen or non-uropathogenic bacteria and the status of whether the sample was retained or removed from analysis.

| Isolate organism type | Presence of other bacteria from metagenomic reads | Status | Reason for status |
| --- | --- | --- | --- |
| *E. coli* | Other uropathogen (*Klebsiella spp.*) | Removed | Mismatch between culture and Kraken classification |
|  | Other uropathogen (*Klebsiella spp.*) | Removed | Mismatch between culture and Kraken classification |
|  | Other uropathogen (*P. aeruginosa*) | Removed | Mismatch between culture and Kraken classification |
|  | Predominantly non-uropathogen (*G. vaginalis)* | Retained | Predominant uropathogen aligned with expected result |
|  | Other uropathogen (*E. cloacae complex*) | Removed | Mismatch between culture and Kraken classification |
|  | Other uropathogen (*Klebsiella spp.*) | Removed | Mismatch between culture and Kraken classification |
|  | Predominantly other (*G. vaginalis)* | Retained | Predominant uropathogen aligned with expected result |
| *Klebsiella spp.* | Other uropathogen (*E. coli*) | Removed | Mismatch between culture and Kraken classification |
|  | Other uropathogen (*P. aeruginosa*) | Removed | Mismatch between culture and Kraken classification |
|  | Other uropathogen (*E. coli*) | Removed | Mismatch between culture and Kraken classification |
|  | Predominantly non-uropathogen (*Lactobacillus spp.)* | Retained | Predominant uropathogen aligned with expected result |

Table S3: Multilocus sequence type (MLST) prediction for primary urine specimens grouped by genus and MLST.

|  |  |  | Lineage Score (LS) < 0.5 | | Lineage Score (LS) ≥ 0.5 | |  |  |
| --- | --- | --- | --- | --- | --- | --- | --- | --- |
| Organism Type | True MLST | Occurrences (n) | Concordant (%) | Discordant (%) | Concordant (%) | Discordant (%) | MLST present in database | Number of isolates in database |
| *E. coli* | 131 | 16 | 0 | 100 (2) | 100 (14) | 0 | Yes | 27 |
| *E. coli* | 69 | 9 | 50 (1) | 50 (1) | 100 (7) | - | Yes | 10 |
| *E. coli* | 95 | 4 | 0 | 100 (1) | 100 (3) | 0 | Yes | 13 |
| *E. coli* | 73 | 3 | - | - | 100 (3) | 0 | Yes | 16 |
| *E. coli* | 127 | 3 | - | - | 100 (3) | 0 | Yes | 5 |
| *E. coli* | 1193 | 6 | 100 (5) | 0 | 0 | 100 (1) | Yes | 14 |
| *E. coli* | 10 | 1 | - | - | 0 | 100 (1) | Yes | 1 |
| *E. coli* | 12 | 3 | 0 | 100 (1) | 100 (2) | 0 | Yes | 4 |
| *E. coli* | 38 | 1 | 100 (1) | 0 | - | - | Yes | 5 |
| *E. coli* | 70 | 2 | 0 | 100 (2) | - | - | No | - |
| *E. coli* | 141 | 1 | - | - | - | 100(1) | Yes | 2 |
| *E. coli* | 193 | 1 | - | 100 (1) | - | - | No | - |
| *E. coli* | 196 | 1 | - | - | - | 100 (1) | No | - |
| *E. coli* | 349 | 2 | 0 | 100 (1) | 0 | 100 (1) | Yes | 1 |
| *E. coli* | 404 | 1 | 0 | 100 (1) | - | - | Yes | 3 |
| *E. coli* | 655 | 1 | 0 | 100 (1) | - | - | No | - |
| *E. coli* | 746 | 1 | 100 (1) | 0 | - | - | Yes | 1 |
| *E. coli* | 998 | 1 | 100 (1) | 0 | - | - | Yes | 1 |
| *E. coli* | 1163 | 2 | - | - | 0 | 100 (2) | No | - |
| *E. coli* | 2556 | 1 | - | - | 0 | 100 (1) | No | - |
| *E. coli* | 2617 | 1 | 0 | 100 (1) | - | - | No | - |
| *E. coli* | 3580 | 1 | - | - | 0 | 100 (1) | No | - |
| *E. coli* | 12159 | 1 | - | - | 0 | 100 (1) | No | - |
| *E. coli* | 12261 | 1 | 0 | 100 (1) | - | - | No | - |
| *Klebsiella* spp. | 45 | 3 | - | - | 33.3 (1) | 66.6 (2) | Yes | 2 |
| *Klebsiella* spp. | 34 | 2 | 0 | 100 (1) | 0 | 100 (1) | Yes | 1 |
| *Klebsiella* spp. | 36 | 1 | - | - | 0 | 100 (1) | Yes | 1 |
| *Klebsiella* spp. | 2004 | 1 | - | - | 0 | 100 (1) | Yes | 1 |
| *Klebsiella* spp. | 194 | 1 | 0 | 100 (1) | - | - | No | - |
| *Klebsiella* spp. | 231 | 1 | - | - | 0 | 100 (1) | Yes | 1 |
| *Klebsiella* spp. | 322 | 1 | 0 | 100 (1) | - | - | No | - |
| *Klebsiella* spp. | 358 | 1 | 0 | 100 (1) | - | - | No | - |
| *Klebsiella* spp. | 412 | 1 | - | - | 0 | 100 (1) | No | - |
| *Klebsiella* spp. | 515 | 1 | 0 | 100 (1) | - | - | No | - |
| *Klebsiella* spp. | 867 | 1 | 0 | 100 (1) | - | - | No | - |
| *Klebsiella* spp. | 2217 | 1 | 0 | 100 (1) | - | - | No | - |
| *Klebsiella* spp. | 2248 | 1 | - | - | 0 | 100 (1) | No | - |

Concordant: the predicted MLST for the best match from RASE matches the true MLST. Not concordant: the predicted MLST for the best match does not match the true MLST.

Table S4: Additional test characteristics (sensitivity, specificity, positive likelihood ratio, and negative likelihood ratio) for each organism type (*E. coli* and *Klebsiella spp.*), stratifying condition, and antibiotic. Stratifying conditions include no stratification (none), stratification by susceptibility scores above 0.6 and below 0.4 (SS), stratification for lineage scores above 0.5 (LS), and stratifying both by susceptibility scores above 0.6 and below 0.4 and lineage scores above 0.5 (SS+LS).

|  |  |  |  |  | Test Characteristic | | | |  |  |  |
| --- | --- | --- | --- | --- | --- | --- | --- | --- | --- | --- | --- |
| Organism Type | Total Susceptibilities | Filtering Condition | Antibiotic | Susceptibilities lost | Sensitivity | Specificity | Positive Likelihood Ratio | Negative Likelihood Ratio | Pre-test Probability | Absolute decrease in  probability of susceptibility with RASE-predicted non-susceptible (%) | Absolute increase in probability of  susceptibility with RASE-predicted susceptible (%) |
| *E.coli* | 64 | None | AMC | 0 | 0.48 (0.22,0.74) | 0.6 (0.39,0.81) | 1.21 (0.69, 2.10) | 0.86 (0.53, 1.40) | 0.45 | -8.05 | 10.3 |
|  | 64 |  | TZP | 0 | 0.58 (0.39,0.77) | 0.52 (0.23,0.82) | 1.22 (0.73, 2.04) | 0.80 (0.46, 1.39) | 0.67 | -7.62 | 6.31 |
|  | 64 |  | CFZ | 0 | 0.38 (0.09,0.67) | 0.74 (0.58,0.91) | 1.48 (0.71, 3.06) | 0.84 (0.54, 1.30) | 0.45 | -9.72 | 21.4 |
|  | 64 |  | CRO | 0 | 0.64 (0.47,0.82) | 0.53 (0.22,0.84) | 1.36 (0.81, 2.29) | 0.68 (0.37, 1.23) | 0.70 | -12.5 | 8.54 |
|  | 61 |  | MEM | 0 | 0.79 (0.67,0.9) | N/A | NA (NA, NA) | NA (NA, NA) | 1.00 | 0 | 0 |
|  | 64 |  | GEN | 0 | 0.76 (0.63,0.88) | 0.67 (0.21,1.13) | 2.28 (0.73, 7.12) | 0.36 (0.14, 0.91) | 0.91 | -14.2 | 5.55 |
|  | 64 |  | CIP | 0 | 0.77 (0.61,0.93) | 0.69 (0.49,0.89) | 2.49 (1.40, 4.40) | 0.33 (0.16, 0.67) | 0.55 | -47.8 | 37.1 |
|  | 64 |  | NIT | 0 | 0.94 (0.88,1) | 0 | 0.94 (0.88, 1.00) | Inf (Inf, Inf) | 0.98 | 1.59 | -0.11 |
|  | 64 |  | SXT | 0 | 0.52 (0.32,0.72) | 0.61 (0.32,0.9) | 1.34 (0.71, 2.55) | 0.78 (0.45, 1.36) | 0.72 | -7.25 | 7.71 |
|  | 64 | SS | AMC | 14 | 0.57 (0.3,0.83) | 0.59 (0.35,0.83) | 1.39 (0.78, 2.48) | 0.73 (0.40, 1.34) | 0.46 | -16.4 | 17.8 |
|  | 64 |  | TZP | 15 | 0.73 (0.55,0.91) | 0.38 (-0.01,0.76) | 1.16 (0.74, 1.80) | 0.73 (0.35, 1.53) | 0.67 | -10.9 | 4.81 |
|  | 64 |  | CFZ | 10 | 0.41 (0.09,0.73) | 0.72 (0.54,0.9) | 1.46 (0.69, 3.07) | 0.82 (0.50, 1.34) | 0.41 | -11.4 | 22.7 |
|  | 64 |  | CRO | 12 | 0.74 (0.57,0.9) | 0.43 (0.03,0.83) | 1.29 (0.79, 2.11) | 0.61 (0.29, 1.30) | 0.73 | -14.5 | 6.43 |
|  | 61 |  | MEM | 5 | 0.84 (0.74,0.95) | N/A | NA (NA, NA) | NA (NA, NA) | 1.00 | 0 | 0 |
|  | 64 |  | GEN | 16 | 0.8 (0.68,0.93) | 0.5 (-0.48,1.48) | 1.61 (0.40, 6.48) | 0.39 (0.09, 1.76) | 0.96 | -6.09 | 1.60 |
|  | 64 |  | CIP | 17 | 0.82 (0.67,0.96) | 0.63 (0.38,0.87) | 2.18 (1.27, 3.75) | 0.29 (0.13, 0.67) | 0.58 | -50.7 | 29.6 |
|  | 64 |  | NIT | 17 | 1 (1,1) | 0 | 1 (1, 1) | NA (NA, NA) | 0.98 | NA | 0 |
|  | 64 |  | SXT | 11 | 0.56 (0.36,0.76) | 0.5 (0.1,0.9) | 1.12 (0.60, 2.10) | 0.88 (0.45, 1.70) | 0.77 | -3.05 | 2.52 |
|  | 64 | LS | AMC | 25 | 0.65 (0.36,0.93) | 0.59 (0.32,0.86) | 1.58 (0.86, 2.92) | 0.60 (0.28, 1.29) | 0.44 | -27.6 | 26.2 |
|  | 64 |  | TZP | 25 | 0.75 (0.55,0.95) | 0.6 (0.28,0.92) | 1.88 (0.97, 3.63) | 0.42 (0.18, 0.98) | 0.62 | -35 | 21.9 |
|  | 64 |  | CFZ | 25 | 0.5 (0.17,0.83) | 0.71 (0.49,0.94) | 1.75 (0.77, 3.67) | 0.7 (0.37, 1.31) | 0.46 | -18.8 | 30 |
|  | 64 |  | CRO | 25 | 0.8 (0.62,0.98) | 0.57 (0.23,0.91) | 1.87 (0.99, 3.53) | 0.35 (0.14, 0.90) | 0.64 | -40 | 20 |
|  | 61 |  | MEM | 25 | 0.84 (0.72,0.97) | N/A | NA (NA, NA) | NA (NA, NA) | 1.00 | 0 | 0 |
|  | 64 |  | GEN | 25 | 0.92 (0.82,1.01) | 1 (1,1) | Inf (NA, Inf) | 0.08 (NA, NA) | 0.92 | -45.8 | 8.33 |
|  | 64 |  | CIP | 25 | 0.87 (0.72,1.02) | 0.75 (0.51,1) | 3.48 (1.47, 8.25) | 0.17 (0.05, 0.56) | 0.59 | -66.1 | 41.3 |
|  | 64 |  | NIT | 25 | 0.95 (0.88,1.02) | N/A | NA (NA, NA) | NA (NA, NA) | 1.00 | 0 | 0 |
|  | 64 |  | SXT | 25 | 0.63 (0.4,0.86) | 0.5 (0.1,0.9) | 1.26 (0.67, 2.38) | 0.74 (0.35, 1.57) | 0.69 | -9.72 | 6.76 |
|  | 64 | SS+LS | AMC | 34 | 0.71 (0.43,0.99) | 0.63 (0.32,0.93) | 1.91 (0.93, 3.89) | 0.46 (0.18, 1.20) | 0.47 | -38.8 | 33.9 |
|  | 64 |  | TZP | 33 | 0.85 (0.68,1.02) | 0.46 (0.02,0.89) | 1.56 (0.88, 2.76) | 0.33 (0.10, 1.10) | 0.65 | -41.9 | 14.6 |
|  | 64 |  | CFZ | 31 | 0.5 (0.13,0.87) | 0.68 (0.43,0.94) | 1.58 (0.68, 3.68) | 0.73 (0.37, 1.46) | 0.42 | -17.5 | 26.9 |
|  | 64 |  | CRO | 31 | 0.83 (0.66,1) | 0.5 (0.06,0.94) | 1.65 (0.87, 3.16) | 0.35 (0.12, 1.03) | 0.70 | -36.2 | 13.6 |
|  | 61 |  | MEM | 30 | 0.94 (0.86,1.02) | N/A | NA (NA, NA) | NA (NA, NA) | 1.00 | 0 | 0 |
|  | 64 |  | GEN | 37 | 0.96 (0.89,1.04) | N/A | NA (NA, NA) | NA (NA, NA) | 1.00 | 0 | 0 |
|  | 64 |  | CIP | 30 | 0.95 (0.86,1.05) | 0.69 (0.39,0.99) | 3.10 (1.36, 7.04) | 0.07 (0.01, 0.50) | 0.62 | -83.8 | 34.9 |
|  | 64 |  | NIT | 28 | 1 (1,1) | N/A | NA (NA, NA) | NA (NA, NA) | 1.00 | NA | 0 |
|  | 64 |  | SXT | 34 | 0.73 (0.51,0.95) | 0.38 (-0.17,0.92) | 1.16 (0.64, 2.11) | 0.73 (0.28, 1.92) | 0.73 | -9.09 | 3.90 |
| *Klebsiella* spp. | 16 | None | AMC | 0 | 0.39 (-0.04,0.81) | 1 (1,1) | Inf (NA, Inf) | 0.62 (NA, NA) | 0.81 | -10.5 | 23.1 |
|  | 16 |  | TZP | 0 | 0.54 (0.17,0.91) | 1 (1,1) | Inf (NA, Inf) | 0.46 (NA, NA) | 0.81 | -17.6 | 23.1 |
|  | 16 |  | CFZ | 0 | 0.62 (0.28,0.95) | 1 (1,1) | Inf (NA, Inf) | 0.39 (NA, NA) | 0.81 | -23.1 | 23.1 |
|  | 16 |  | CRO | 0 | 0.77 (0.51,1.03) | 1 (1,1) | Inf (NA, Inf) | 0.23 (NA, NA) | 0.81 | -38.5 | 23.1 |
|  | 16 |  | MEM | 0 | 0.81 (0.6,1.02) | N/A | NA (NA, NA) | NA (NA, NA) | 1.00 | 0 | 0 |
|  | 16 |  | GEN | 0 | 0.93 (0.8,1.06) | 1 (1,1) | Inf (NA, Inf) | 0.07 (NA, NA) | 0.94 | -46.7 | 6.67 |
|  | 16 |  | CIP | 0 | 0.85 (0.63,1.06) | 0.33 (-0.59,1.26) | 1.27 (0.55, 2.92) | 0.46 (0.08, 2.54) | 0.81 | -18.0 | 4.14 |
|  | 16 |  | SXT | 0 | 0.75 (0.47,1.03) | 0.5 (-0.19,1.19) | 1.5 (0.53, 4.21) | 0.5 (0.13, 2.00) | 0.75 | -20 | 9.09 |
|  | 16 | SS | AMC | 4 | 0.56 (0.12,0.99) | 1 (1,1) | Inf (NA, Inf) | 0.44 (NA, NA) | 0.75 | -23.8 | 33.3 |
|  | 16 |  | TZP | 4 | 0.67 (0.29,1.04) | 1 (1,1) | Inf (NA, Inf) | 0.33 (NA, NA) | 0.75 | -33.3 | 33.3 |
|  | 16 |  | CFZ | 0 | 0.62 (0.28,0.95) | 1 (1,1) | Inf (NA, Inf) | 0.39 (NA, NA) | 0.81 | -23.1 | 23.1 |
|  | 16 |  | CRO | 0 | 0.77 (0.51,1.03) | 1 (1,1) | Inf (NA, Inf) | 0.23 (NA, NA) | 0.81 | -38.5 | 23.1 |
|  | 16 |  | MEM | 0 | 0.81 (0.6,1.02) | N/A | NA (NA, NA) | NA (NA, NA) | 1.00 | 0 | 0 |
|  | 16 |  | GEN | 0 | 0.93 (0.8,1.06) | 1 (1,1) | Inf (NA, Inf) | 0.07 (NA, NA) | 0.94 | -46.7 | 6.67 |
|  | 16 |  | CIP | 0 | 0.85 (0.63,1.06) | 0.33 (-0.59,1.26) | 1.27 (0.55, 2.92) | 0.46 (0.08, 2.54) | 0.81 | -18.0 | 4.14 |
|  | 16 |  | SXT | 0 | 0.75 (0.47,1.03) | 0.5 (-0.19,1.19) | 1.5 (0.53, 4.21) | 0.5 (0.13, 2.00) | 0.75 | -20 | 9.09 |
|  | 16 | LS | AMC | 7 | 0.38 (-0.17,0.92) | 1 (1,1) | Inf (NA, Inf) | 0.63 (NA, NA) | 0.89 | -6.25 | 12.5 |
|  | 16 |  | TZP | 7 | 0.5 (0.01,0.99) | 1 (1,1) | Inf (NA, Inf) | 0.5 (NA, NA) | 0.89 | -10 | 12.5 |
|  | 16 |  | CFZ | 7 | 0.75 (0.4,1.1) | 1 (1,1) | Inf (NA, Inf) | 0.25 (NA, NA) | 0.89 | -25 | 12.5 |
|  | 16 |  | CRO | 7 | 0.88 (0.63,1.12) | 1 (1,1) | Inf (NA, Inf) | 0.13 (NA, NA) | 0.89 | -43.8 | 12.5 |
|  | 16 |  | MEM | 7 | 0.89 (0.67,1.11) | N/A | NA (NA, NA) | NA (NA, NA) | 1.00 | 0 | 0 |
|  | 16 |  | GEN | 7 | 1 (1,1) | 1 (1,1) | Inf (NA, Inf) | 0 (NA, NA) | 0.89 | -100 | 12.5 |
|  | 16 |  | CIP | 7 | 1 (1,1) | 0.33 (-0.59,1.26) | 1.5 (0.67, 3.34) | 0 (0, NA) | 0.67 | -100 | 12.5 |
|  | 16 |  | SXT | 7 | 0.83 (0.51,1.16) | 0.67 (0.01,1.32) | 2.5 (0.49, 12.89) | 0.25 (0.03, 2.08) | 0.67 | -50 | 25 |
|  | 16 | SS+LS | AMC | 11 | 0.75 (0.26,1.24) | 1 (1,1) | Inf (NA, Inf) | 0.25 (NA, NA) | 0.80 | -37.5 | 25 |
|  | 16 |  | TZP | 11 | 0.75 (0.26,1.24) | 1 (1,1) | Inf (NA, Inf) | 0.25 (NA, NA) | 0.80 | -37.5 | 25 |
|  | 16 |  | CFZ | 7 | 0.75 (0.4,1.1) | 1 (1,1) | Inf (NA, Inf) | 0.25 (NA, NA) | 0.89 | -25 | 12.5 |
|  | 16 |  | CRO | 7 | 0.88 (0.63,1.12) | 1 (1,1) | Inf (NA, Inf) | 0.13 (NA, NA) | 0.89 | -43.8 | 12.5 |
|  | 16 |  | MEM | 7 | 0.89 (0.67,1.11) | N/A | NA (NA, NA) | NA (NA, NA) | 1.00 | 0 | 0 |
|  | 16 |  | GEN | 7 | 1 (1,1) | 1 (1,1) | Inf (NA, Inf) | 0 (NA, NA) | 0.89 | -100 | 12.5 |
|  | 16 |  | CIP | 7 | 1 (1,1) | 0.33 (-0.59,1.26) | 1.5 (0.67, 3.34) | 0 (0, NA) | 0.67 | -100 | 12.5 |
|  | 16 |  | SXT | 7 | 0.83 (0.51,1.16) | 0.67 (0.01,1.32) | 2.5 (0.49, 12.89) | 0.25 (0.03, 2.08) | 0.67 | -50 | 25 |

GEN = gentamicin; AMC = amoxicillin-clavulanic acid; CFZ = cefazolin; CRO = ceftriaxone; CIP = ciprofloxacin; MEM = meropenem; NIT = nitrofurantoin; TZP = piperacillin-tazobactam; SXT = trimethoprim-sulfamethoxazole

Table S5: Local reference database (Ottawa, Canada and Toronto, Canada) characteristics, including sample origin location, sample number, unique multilocus sequence types (MLSTs), and phenotypic susceptibility profiles for the evaluated antibiotics.

|  |  |  |  |  |  | % Susceptible per antibiotic | | | | | | | | |
| --- | --- | --- | --- | --- | --- | --- | --- | --- | --- | --- | --- | --- | --- | --- |
| Reference Database | Location | Organism Type | Samples |  | MLSTs Represented | GEN | AMC | CFZ | CRO | CIP | MEM | NIT | TZP | SXT |
| Local | Ottawa, Canada | *E. coli* | 42 |  | 21 | 93 | 79 | 69 | 81 | 64 | 100 | 100 | 77 | 81 |
|  |  | *Klebsiella* *spp*. | 41 |  | 32 | 95 | 83 | 59 | 78 | 85 | 98 | - | 90 | 85 |
| Local | Toronto, Canada | *E. coli* | 106 |  | 40 | 86 | 51 | 57 | 71 | 65 | 100 | 92 | 63 | 58 |
|  |  | *Klebsiella* *spp*. | 90 |  | 74 | 99 | 56 | 46 | 59 | 90 | 97 | - | 51 | 89 |

GEN = gentamicin; AMC = amoxicillin-clavulanic acid; CFZ = cefazolin; CRO = ceftriaxone; CIP = ciprofloxacin; MEM = meropenem; NIT = nitrofurantoin; TZP = piperacillin-tazobactam; SXT = trimethoprim-sulfamethoxazole

Table S6: Reference database characteristics for EuSCAPE database, including the number of samples and susceptibility profiles for the included antibiotics which overlapped with the regional (Ontario, Canada) database.

|  |  |  |  |  | % Susceptible per antibiotic | | | |
| --- | --- | --- | --- | --- | --- | --- | --- | --- |
| Reference Database | Location | Organism Type | Samples | STs Represented | CIP | GEN | MEM | TZP |
| European | Europe | *K. pneumoniae* | 1511 | 241 | 36 | 61 | 79 | 24 |

CIP = ciprofloxacin; GEN = gentamicin; MEM = meropenem; TZP = piperacillin-tazobactam

Table S7: Sequence Type Prediction for Primary Urine Specimens containing *Klebsiella* spp. from Ontario, Canada analyzed using the EuSCAPE *Klebsiella pneumoniae* database.

|  |  | LS < 0.5 | | LS > 0.5 | |
| --- | --- | --- | --- | --- | --- |
| Samples (n) | MLSTs Represented | Concordant  (n, %) | Not concordant (n, %) | Concordant (n, %) | Not concordant (n, %) |
| 16 | 13 | 3 (33.3) | 6 (66.6) | 4 (57.1) | 3 (42.9) |

Table S8: Multilocus sequence type (MLST) prediction for primary urine specimens grouped by genus and MLST when queried using the EuSCAPE *Klebsiella pneumoniae* database.

|  |  | Lineage Score (LS) < 0.5 | | Lineage Score (LS) ≥ 0.5 | |  |  |  |
| --- | --- | --- | --- | --- | --- | --- | --- | --- |
| True MLST | Occurrences (n) | Concordant (n) | Discordant (n) | Concordant  (n) | Discordant  (n) | Present in Canadian databases | MLST present in database | Number of isolates in database |
| 34 | 2 | 0 | 1 | 0 | 1 | Yes | Yes | 3 |
| 36 | 1 | 1 | 0 | - | - | Yes | Yes | 12 |
| 45 | 3 | 2 | 1 | - | - | Yes | Yes | 37 |
| 194 | 1 | 0 | 1 | - | - | No | No | 0 |
| 231 | 1 | - | - | 1 | 0 | Yes | Yes | 1 |
| 322 | 1 | - | - | 1 | 0 | No | Yes | 1 |
| 358 | 1 | - | - | 0 | 1 | No | No | 0 |
| 412 | 1 | 0 | 1 | - | - | No | No | 0 |
| 515 | 1 | 0 | 1 | - | - | No | No | 0 |
| 867 | 1 | - | - | 1 | 0 | No | Yes | 1 |
| 2004 | 1 | - | - | 0 | 1 | Yes | No | 0 |
| 2217 | 1 | - | - | 1 | 0 | No | Yes | 2 |
| 2248 | 1 | 0 | 1 | - | - | No | No | 0 |

**SUPPLEMENTAL FIGURES**


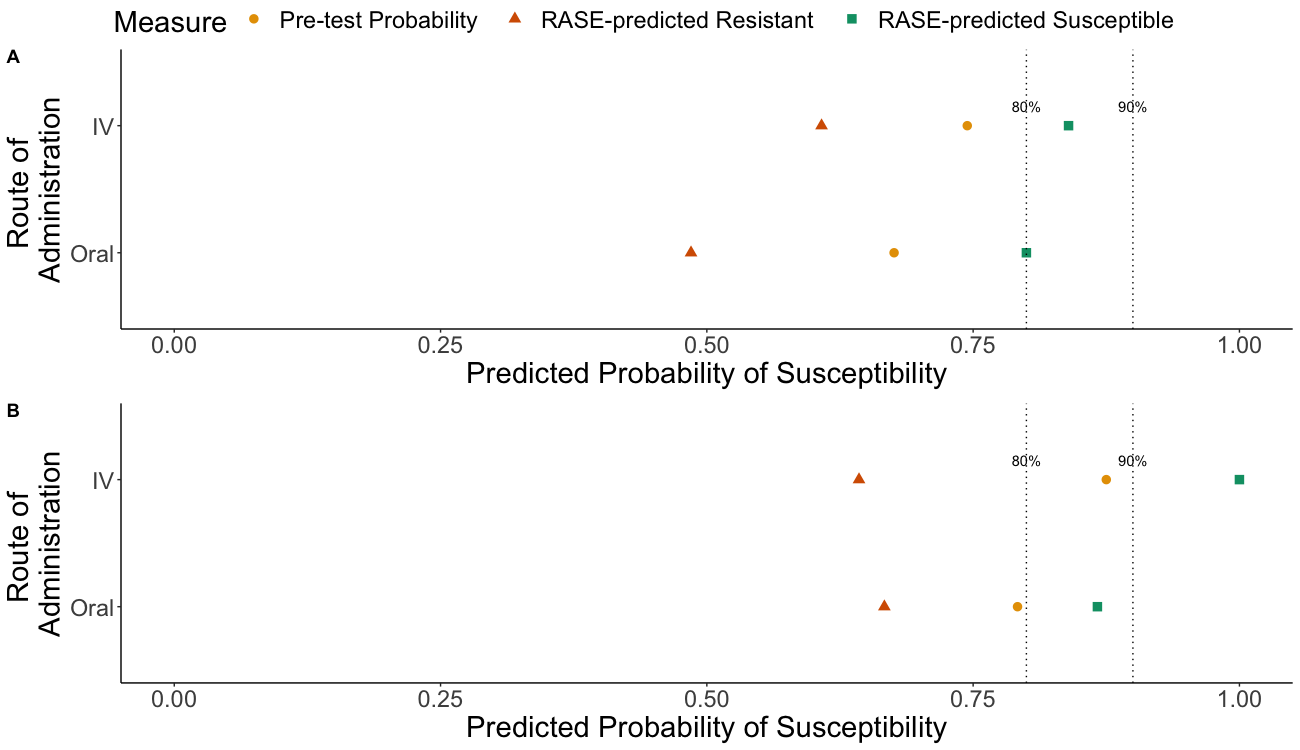


Figure S1: Pre-test probability (yellow circles), RASE-predicted non-susceptible (orange triangles), and RASE-predicted susceptible (green squares) values determined for (A) *E. coli* and (B) *Klebsiella* spp. when antibiotics were split by the typical route of administration (oral vs intravenous (IV))*.* Oral antibiotics were AMC, CIP, NIT, and SXT. IV antibiotics were CFZ, CRO, GEN, MEM, and TZP. Dotted lines at 0.8 and 0.9 represent the low-severity and high-severity disease thresholds respectively, derived from [(10)](https://paperpile.com/c/BqiAWA/x7xvW). Antibiotic short forms used in figure: GEN = gentamicin; AMC = amoxicillin-clavulanic acid; CFZ = cefazolin; CRO = ceftriaxone; CIP = ciprofloxacin; MEM = meropenem; NIT = nitrofurantoin; TZP = piperacillin-tazobactam; SXT = trimethoprim-sulfamethoxazole.

**SUPPLEMENTAL RESULTS**

*Improving Phenotype Prediction Using Confidence Scores*

For each dataset, we found that 64.1%, 37.5%, and 59.2% of samples were concordant for *E. coli*, *Klebsiella spp.,* and the combined data, respectively (Table 3). For both *E. coli* and the combined data, the LR+ was higher for the concordant calls (*E. coli*: 2.77 (95% CI:1.99, 3.86); Combined: 2.73 (95% CI: 1.99, 3.73)) than the discordant calls (*E. coli*: 1.07 (95% CI:0.84, 1.36); Combined: 1.26 (95% CI: 0.99, 1.61)). This is the opposite to what we observed for the LR+ for *Klebsiella spp.,* with the LR+ for concordant calls being 2.38 (95% CI: 0.88, 6.42) and 6.72 (95% CI: 1.05, 42.85) for discordant calls. For both *E. coli* and the combined data, the LR- was lower for the concordant calls (*E. coli*: 0.40 (95% CI: 0.31, 0.52); Combined: 0.41 (95% CI: 0.32, 0.53)) than the discordant calls (*E. coli*: 0.88 (95% CI: 0.62, 1.25); Combined: 0.68 (95% CI: 0.50, 0.94)). Again, this is the opposite to what we observed for the LR- for *Klebsiella spp.,* with the LR- for concordant calls being 0.48 (95% CI: 0.23, 1.01) and 0.29 (95% CI: 0.13, 0.61) for discordant calls. This was done because concordance was generally found to be higher when LS > 0.5, which we believe indicates that LS is mediated, in part, through concordance.

*Evaluating the Performance of City-specific Prediction*

We then used city-specific databases for *E. coli* to predict the susceptibility for city-specific samples (either Ottawa or Toronto) in order to evaluate how well these city-specific databases performed relative to the regional databases (Figure S2). We tested the samples from both Ottawa and Toronto separately against the Ottawa database (Figure S2A and S2B), the Toronto database (Figure S2C and S2D), and the combined database for comparison (Figure S2E and S2F). Using the local Ottawa database with the Ottawa primary specimens (Figure S2A), the values for ampicillin-clavulanic acid, cefazolin, and ceftriaxone are inverted; however, the RASE-predicted susceptible values for sulfamethoxazole-trimethoprim and ciprofloxacin were above the 90% threshold. Using the Toronto primary specimens against the local Ottawa database, while values were in the expected order, none surpassed their pre-test probability by overcoming either the 80% or 90% thresholds (Figure S2B). Using the Toronto-specific database, Ottawa samples obtained RASE-predicted susceptibility probabilities over the 80% threshold for piperacillin-tazobactam and ciprofloxacin, while ceftriaxone was above the 90% threshold (Figure S2C). Toronto samples analyzed with the Toronto database only had a probability of susceptibility over the 90% threshold for gentamicin (Figure S2D). In order to more meaningfully compare the results of the city-specific databases, we also analyzed the results for the city-specific samples using the regional database. In the case of the Ottawa samples (Figure S2E), the probability of susceptibility surpasses the 80% low-severity illness threshold, and ciprofloxacin and sulfamethoxazole-trimethoprim reach the 90% high-severity illness threshold. Compared to the same samples analyzed using the Ottawa-specific database, ceftriaxone susceptibility prediction was improved, and none of the predictive values were inverted. For Toronto-specific samples analyzed with the combined database, we observe that the probability of susceptibility for none of the antibiotics surpasses the pre-test probability and neither the 80% or 90% thresholds (Figure S2F). This is similar to what we observed for the same samples analyzed with the Ottawa-specific local database, however, using the Toronto-specific local database, the probability of susceptibility for gentamicin exceeded the 90% high-severity illness threshold.


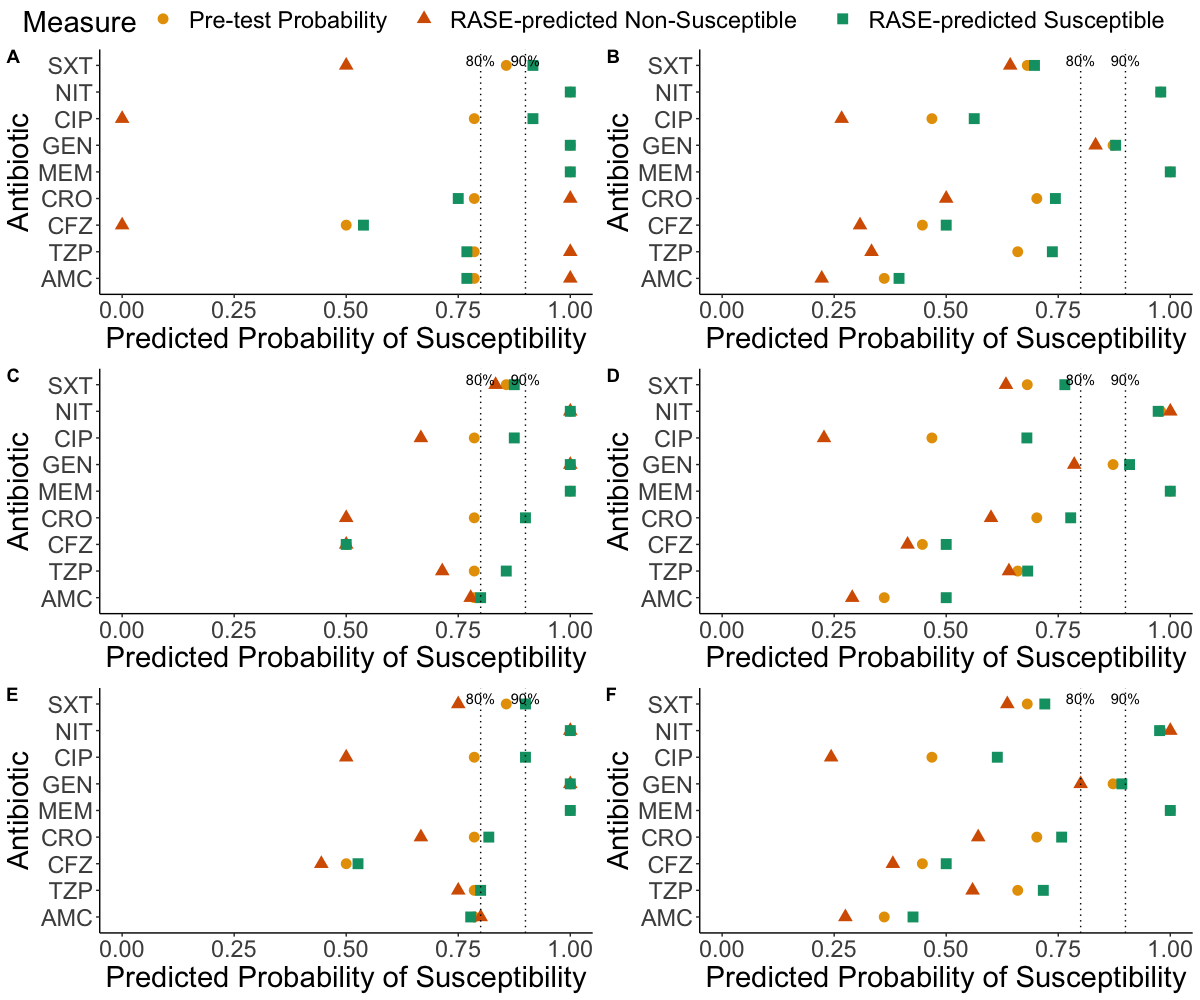


Figure S2: Pre-test probability (yellow circles), RASE-predicted non-susceptible (orange triangles), and RASE-predicted susceptible (green squares) values determined for the overall prediction for each tested antibiotic for *E. coli* using Ottawa- or Toronto-specific local databases. Ottawa specimens were analyzed using (A) an Ottawa-specific local database, (C) a Toronto-specific local database, and (E) the combined regional database. Toronto specimens were analyzed using (B) an Ottawa-specific local database, (D) a Toronto-specific local database, and (F) the combined regional database. Dotted lines at 0.8 and 0.9 represent the low-severity and high-severity disease thresholds respectively [(25)](https://paperpile.com/c/BqiAWA/ZiCHG), derived from [(10)](https://paperpile.com/c/BqiAWA/x7xvW). Antibiotic short forms used in figure: AMC = amoxicillin-clavulanic acid; TZP = piperacillin-tazobactam; CFZ = cefazolin; CRO = ceftriaxone; MEM = meropenem; GEN = gentamicin; CIP = ciprofloxacin; NIT = nitrofurantoin; SXT = trimethoprim-sulfamethoxazole.

*Evaluating the Performance of An International Reference Database*

Finally, we assessed prediction performance by running the reads from Ontario’s samples through a *K. pneumoniae* database constructed in Germany using 1511 isolates from EuSCAPE. This database contains 241 unique MLSTs, and susceptibility was generally lower than those sampled in Ontario for the overlapping antibiotics (Table S6). As described above, we only compared the susceptibility predictions for antibiotics which overlapped with those in the Ontario, Canada database (e.g., gentamicin, ciprofloxacin, meropenem, and piperacillin-tazobactam). The Rase-predicted probabilities are largely not informative for meropenem and piperacillin-tazobactam (Figure S3). However, the RASE-predicted susceptible values for gentamicin and ciprofloxacin are well separated. These data suggest ciprofloxacin could be rescued for empiric use in high severity conditions given that the RASE-predicted susceptible results is over the 90% threshold For both agents, a RASE-predicted non-susceptible result is useful for defining a infection that has a sufficient risk of non-susceptible to necessitate choosing an alternative agent based on the minimum treatment threshold of 80%. ST concordance for the samples was also analyzed, and are similar to what was found with the regional database (Table S3). Notably, concordance was also improved when samples had a higher (i.e., above 0.5) lineage score.


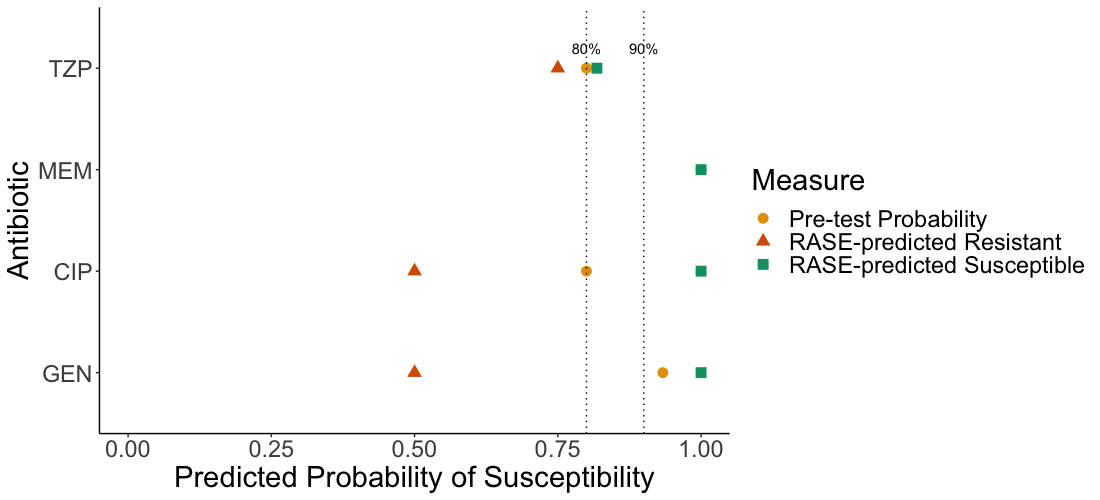


Figure S3: Pre-test probability (yellow circles), RASE-predicted non-susceptible (orange triangles), and RASE-predicted susceptible (green squares) values determined for the overall prediction for each tested antibiotic for *Klebsiella* spp collected in Ontario, Canada and run against the *Klebsiella pneumoniae* database constructed using EuSCAPE isolates *.* Dotted lines at 0.8 and 0.9 represent the low-severity and high-severity disease thresholds respectively, derived from [(10)](https://paperpile.com/c/BqiAWA/x7xvW). Antibiotic short forms used in figure: GEN = gentamicin; CIP = ciprofloxacin; MEM = meropenem; TZP = piperacillin-tazobactam.

**SUPPLEMENTAL MATERIALS**

*Solutions and Reagent Preparation*

PBS was made by diluting a 10X stock (1.37M NaCl, 27mM KCl, 100mM Na_2_HPO_4_, 18mM KH_2_PO_4_) 1:10 using distilled water, and then sterilized by autoclave. A solution of 5% saponin was made by mixing 5g of saponin with 80mL of water, and the volume was adjusted to 100mL. HL-SAN buffer was made to a final concentration of 0.5M NaCl and 100mM MgCl_2_ in water and sterilized.

*Isolate Reference Database Generation (Expanded)*

At participating institutions, Gram-negative isolates from select organism types (*E.coli* and *Klebsiella spp.*) from clinical specimens (any source) originating from critically ill patients were collected prospectively across a six month period. These isolates were sequenced (short-read approaches) and clinically derived antibiotic susceptibility phenotype were collected as meta-data. Following short-read sequencing, the resultant sequencing files (Fastq) were assessed for quality using FastQC v0.11.9 (<https://www.bioinformatics.babraham.ac.uk/projects/fastqc/>), and reads were then trimmed using Trimmomatic (v0.36) with the following parameters: LEADING:10 TRAILING:10 SLIDINGWINDOW:4:15 MINLEN:50 (Bolger et al., 2014). Following quality control and trimming, sequences were assembled using SPAdes (Prjibelski et al., 2020). Assembled sequences along with antibiotic susceptibility phenotype, phylogeny (Katz et al., 2019), and genotypic multi-locus susceptibility phenotyping (Seemann, n.d.) were used to generate organism type specific RASE k-mer reference databases (Břinda et al., 2020). Databases were created using a *k*-mer length of 18.

*Human genomic material depletion and bacterial DNA extraction:*

Samples were thawed from -70°C and brought to room temperature prior to processing, with 1.5 mL of urine being transferred to a sterile 1.5 mL tube. The samples were centrifuged at 12000*g* for 5 minutes, the supernatant was discarded, and 1 mL of 1X PBS was added to the tube without disturbing the pellet. This was repeated twice. Following a final spin at 12000*g*, the pellet was resuspended in 200 µL of 1X PBS and 200 µL of 5% saponin. These were incubated at 37°C for 15 minutes, and following incubation, 350 µL of water was added, and 12 µL of 5M NaCl was added after 30 seconds. The tubes were centrifuged at 6000*g* for 5 minutes, the supernatant discarded, and the pellets resuspended in 100 µL 1X PBS. Following resuspension, 100 µL of HL-SAN buffer and 10 µL of HL-SAN DNase (ArcticZymes) were added to the tubes, which were then vortexed briefly, and incubated at 37°C for 15 minutes. To remove the DNase and cellular and genomic debris, 800 µL of 1X PBS was added to each tube, and these were centrifuged for 3 minutes at 6000*g*. The supernatant was discarded, and 1 mL of 1X PBS was added while leaving the pellet undisturbed prior to a second 6000*g* spin for 3 minutes. The supernatant was removed, and the pellet was used directly for genomic extractions using the Gram-negative bacteria protocol for the GeneJet Genomic DNA Purification Kit (ThermoFisher K0722) with 50 µL elution volumes. For each set of genomic extractions, a single negative control was used, substituting sterile water for urine and processing as above.

*Using RASE for Susceptibility Predictions*

In order to use RASE, we first had to create the databases to use for prediction. This was done using the RASE DB Skeleton software (Břinda et al, 2020). Databases are constructed using known isolates for the organism type of interest and require the following: whole genome assembly for each of the isolates to include in the database in fasta format, a genetic tree for the included isolates made using mashtree (Katz et al., 2019), as well as metadata for each of the isolates. This metadata includes both information for the multiple locus sequence type (MLST), as well as the susceptibility result for each of the antibiotics that are to be included in the database; for our databases, we included the susceptibility or non-susceptibility determination, as well as an interpreted “MIC” from this data (susceptible calls were given an “MIC” of 0, while non-susceptible calls were given an “MIC” of 999). The susceptibility cutoffs used for creating the databases followed the EUCAST breakpoints (EUSCAST, 2024). Following the incorporation of these data into the database skeleton, the databases were created following the steps described in the RASE documentation using k-mer size of 18bp (Břinda et al, 2020).

Once the databases were constructed and the samples sequenced, including confirmation of pathogen organism type using Kraken, sample read fastq files were analysed using the RASE pipeline software (Břinda et al, 2020). The software was used as described in the documentation; however, a brief description of the method follows: the constructed database for the organism type of interest was moved into the appropriate folder for the database, and the reads to be analysed were moved into the “Reads” folder. Following this, the program was run as documented, and the sample outputs required for further analysis were retrieved in the “Prediction” folder.

RASE works by taking a nanopore reads, splitting it into *k*-mers, and matching it to the database using ProPhyle (Břinda et al, 2020). As more *k-*mers and reads are analysed, the weight for the reference database isolates are updated. Upon completion of analysing all reads, the lineage containing the best match with the highest proportion of matched *k*-mers is determined, as well as the lineage containing the second-best match. A lineage score (LS) is found using the following calculation: LS = 2f/(f+t)-1, where f is the weight of the isolate with the best match in the best-matched lineage and t is the weight of the isolate with the best match in the alternative lineage. Values range from 0 to 1, with a score of 0 indicating the predicted and alternative lineages were equally matched, while a score of 1 indicates complete matching to the predicted lineage. Lower scores, particularly those below 0.5, indicate that lineage assignment may need further investigation. A susceptibility score (SS) is calculated as SS= s/(s+r), where the values are the weights of the susceptible (s) and non-susceptible (r) best matches within the predicted lineage. Scores range from 0 to 1, with both samples being non-susceptible or susceptible, respectively. Scores above 0.5 indicate a probably susceptible phenotype, and scores below 0.5 indicate a probably non-susceptible phenotype. Scores of 0.5 mean that the susceptible and non-susceptible best matches are equally weighted; however, scores between 0.4 and 0.6 indicate lower-confidence calls, which may further reflect a recent emergence of susceptibility or non-susceptibility in that lineage.

*Imputation*

We performed imputation of selected antibiotic susceptibility phenotypes for both the reference isolates and primary urinary specimens. To align with laboratory practices, for isolates that had a non-susceptible phenotype returned for ceftriaxone, we changed any susceptible results for the penicillin-β-lactam inhibitor combinations (amoxicillin-clavulanic acid, piperacillin-tazobactam) to non-susceptible.

*Data Availability*

RASE reference databases and meta-data are available on Zenodo (DOI: [10.5281/zenodo.11042579 and](https://doi.org/10.5281/zenodo.11042579) [10.5281/zenodo.11121227)](https://doi.org/10.5281/zenodo.11121227). Isolate sequence data are also available on Zenodo (DOI: 10.5281/zenodo.11192433 and 10.5281/zenodo.11193854).

*Data Analysis - Test Characteristics*

For each sample, predictions were classified as true positive (predicted susceptible by RASE and clinical phenotype susceptible), false positive (predicted susceptible by RASE and clinical phenotype non-susceptible), true negative (predicted non-susceptible by RASE and clinical phenotype non-susceptible), and false negative (predicted non-susceptible by RASE and clinical phenotype susceptible) for each antibiotic.

To compute our test statistics, we created a pipeline in R to automatically calculate these values and produce the graphical representations (Supplemental Materials). True positives (TP) were assigned when both the true and predicted susceptibility calls were S, false positives (FP) when the predicted call was S but truly R, false negatives (FN) when the predicted call was R but truly S, and true negatives (TN) when both the true and predicted calls were R. Please see below for the equations for positive predictive values (PPV), negative predictive values (NPV), pre-test probability, sensitivity, specificity, negative likelihood ratio (LR-), and positive likelihood ratio (LR+).

*Positive predictive value (PPV)* = $\frac{TP\left( n \right)}{\left( TP\left( n \right)+FP\left( n \right) \right)}$

*Negative predictive value (NPV)* = $\frac{TN\left( n \right)}{\left( TN\left( n \right)+FN\left( n \right) \right)}$ ; note: use 1-NPV

*Pre-test probability* = $\frac{\left( TP\left( n \right)+FN\left( n \right) \right)}{\left( TP\left( n \right)+FP\left( n \right)+FN\left( n \right)+TN\left( n \right) \right)}$

*Sensitivity* = $\frac{TP\left( n \right)}{\left( TP\left( n \right)+FN\left( n \right) \right)}$

*Specificity* =$\frac{TN\left( n \right)}{}$

*Positive likelihood ratio (LR+)* = $\frac{Sensitivity}{1-Specificity}$

*Negative likelihood ratio (LR-)* = $\frac{1-Sensitivity}{Specificity}$

We also calculated the 95% confidence intervals (CI) for these values using the following equations:

*CI_PPV_* = $PPV\pm1.96\times\sqrt{\frac{PPV\times\left( 1-PPV \right)}{\left( TP\left( n \right)+FP\left( n \right) \right)}}$

*CI_NPV_* = $NPV\pm1.96\times\sqrt{\frac{NPV\times\left( 1-NPV \right)}{\left( TN\left( n \right)+FN\left( n \right) \right)}}$

*CI_Sensitivity_* = $Sensitivity\pm1.96\times\sqrt{\frac{Sensitivity\left( 1-Sensitivity \right)}{TP\left( n \right)+FN\left( n \right)}}$

*CI_Specificity_* = $Specificity\pm1.96\times\sqrt{\frac{Specificity\left( 1-Specificity \right)}{FP\left( n \right)+TN\left( n \right)}}$

*CI_Positive Likelihood Ratio (LR+)_* = $e^{ln}$

*CI_Negative Likelihood Ratio (LR-)_* = $e^{ln}$

References for Supplement:

Bolger AM, Lohse M, Usadel B. Trimmomatic: a flexible trimmer for Illumina sequence data. Bioinformatics. 2014 Aug 1;30(15):2114–20.

Břinda K, Callendrello A, Ma KC, MacFadden DR, Charalampous T, Lee RS, et al. Rapid inference of antibiotic resistance and susceptibility by genomic neighbour typing. Nat Microbiol. 2020 Mar;5(3):455–64.

EUCAST. Breakpoint tables for interpretation of MICs and zone diameters. Version 14.0, 2024. http://www.eucast.org.

Katz LS, Griswold T, Morrison SS, Caravas JA, Zhang S, den Bakker HC, et al. Mashtree: a rapid comparison of whole genome sequence files. Journal of Open Source Software. 2019 Dec 10;4(44):1762.

Prjibelski A, Antipov D, Meleshko D, Lapidus A, Korobeynikov A. Using SPAdes De Novo Assembler. Curr Protoc Bioinformatics. 2020 Jun 1;70(1):e102.

Seemann T, mlst Github https://github.com/tseemann/mlst. [cited 2023 Nov 7]
